# A Topical Review on Enabling Technologies for the Internet of Medical Things: Sensors, Devices, Platforms, and Applications

**DOI:** 10.3390/mi15040479

**Published:** 2024-03-30

**Authors:** Md. Shamsul Arefin, Mohammed Mostafizur Rahman, Md. Tanvir Hasan, Mufti Mahmud

**Affiliations:** 1 Department of Electrical and Electronic Engineering (EEE), Bangladesh University of Business & Technology, Dhaka 1216, Bangladesh; shamsul.a@bubt.edu.bd; 2 Department of Mathematics, American International University-Bangladesh, Dhaka 1229, Bangladesh; mostafiz.math@aiub.edu; 3 Department of Electrical and Electronic Engineering (EEE), Jashore University of Science & Technology, Jashore 7408, Bangladesh; mth@just.edu.bd; 4 Department of Electrical Engineering, University of South Carolina, Columbia, SC 29208, USA; 5 Department of Computer Science, Nottingham Trent University, Nottingham NG11 8NS, UK; 6 Computing and Informatics Research Centre, Nottingham Trent University, Nottingham NG11 8NS, UK; 7 Medical Technologies Innovation Facility, Nottingham Trent University, Nottingham NG11 8NS, UK

**Keywords:** IoMT, sensors, IoT platform, AI, data management, COVID-19

## Abstract

The Internet of Things (IoT) is still a relatively new field of research, and its potential to be used in the healthcare and medical sectors is enormous. In the last five years, IoT has been a go-to option for various applications such as using sensors for different features, machine-to-machine communication, etc., but precisely in the medical sector, it is still lagging far behind compared to other sectors. Hence, this study emphasises IoT applications in medical fields, Medical IoT sensors and devices, IoT platforms for data visualisation, and artificial intelligence in medical applications. A systematic review considering PRISMA guidelines on research articles as well as the websites on IoMT sensors and devices has been carried out. After the year 2001, an integrated outcome of 986 articles was initially selected, and by applying the inclusion–exclusion criterion, a total of 597 articles were identified. 23 new studies have been finally found, including records from websites and citations. This review then analyses different sensor monitoring circuits in detail, considering an Intensive Care Unit (ICU) scenario, device applications, and the data management system, including IoT platforms for the patients. Lastly, detailed discussion and challenges have been outlined, and possible prospects have been presented.

## 1. Introduction

The Internet has revolutionised human life by facilitating information exchange across different devices. This has been possible by allowing them to be connected together, forming a new network known as the Internet of Things (IoT). Literally, ‘everything’ that is internet-enabled is getting connected to this network to produce smarter and better solutions for end users. The IoT is established through the connection of diverse devices to the Internet, allowing for interconnectivity among these devices. In recent days, IoT has been widely used for commercial and emerging technology use cases, including connected homes, hospitals, aerospace, communications, and other digitised spaces [1,2]. Even though the international IoT market is projected to be approximately $14.4 trillion by 2022 [3], this idea is still new to many consumer groups. IoT is comprised of a combination of technologies, including wireless communication, embedded systems, machine learning, and real-time analytics. As a technology, IoT foresees a future where digital and physical mediums are linked using proper data communication to facilitate various applications and use cases in the real world [4,5].

The Internet of Medical Things (IoMT) or Medical Internet of Things (MIoT) is a connected system combining software applications with medical devices that connect to different healthcare IT systems. IoMT ensures the optimisation of healthcare delivery by establishing a secure connection between patients, healthcare service providers, and medical devices, thereby creating a reliable environment for healthcare service provisioning. Hence, it has emerged as a dependable technology to assist medical personnel in managing patient information and delivering secure digitised healthcare services. The healthcare IoT market has been anticipated to jump from USD 32.47 billion in 2015 to USD 163.24 billion by 2020, at a compound annual growth rate (CAGR) of 38.1% from 2015 to 2020 [6]. Also predicted to witness remarkable expansion, the global IoT in the healthcare market is expected to grow significantly from USD 127.7 billion in 2023 to USD 289.2 billion in 2028. This growth is projected to occur at a CARG of 17.8% throughout the forecast period [7]. Moreover, IoT brings forth a multitude of applications and use cases in the medical industry, offering valuable aid to patients, families, and medical practitioners. In terms of connectivity and connected networks in the healthcare area, approximately 20 billion devices are likely to be in use over the next few years [1,2,6].

To ensure comprehensive patient data collection and offer advanced solutions, contemporary medical devices must include chips or cutting-edge technological features. Remote health management, managing various diseases and conditions, fitness programmes, patient monitoring at home, enduring disease conditions, and elderly patient care and monitoring are imperative use cases. Advancement in medical devices, mainly in home-use cases in the diagnosing and imaging sectors, plays a crucial role and is one of the vital technology components in the market. With rising demands and advancements in technology, medical device companies are compelled to incorporate technology in order to offer a greater variety of distinctive features. As of now, devices containing sensors, controllers, wireless capability, upgraded firmware, and remote monitoring meet various healthcare trends and demands. A connected healthcare solution ensures a fast and dependable information flow to healthcare professionals, granting convenient accessibility and equipping medical workers with tools to analyse patient data and determine appropriate recommendations. Hence, the patient gets a better-quality home care facility by keeping in touch with the doctors. Connected healthcare systems can also assist in identifying the stages of common diseases, for example, hypertension, asthma, and others. Also, the incorporation of modern technology and the need to swiftly enter the market prompt device manufacturers to meet the requirements [1]. Therefore, health monitoring or Medical IoT devices vary from cardiac monitoring to electronic wristbands, smart beds, medical devices, and others. The primary aim is to reduce manual work and generate opportunities for higher precision, efficiency, and profits by continuously incorporating IoT features into healthcare devices [8]. Moreover, this system is much safer and easier to use for patients, and it permits clinicians to work on the data that the devices generate and provide much better information on patients’s wellbeing [2].

Subsequently, the flexibility of gathering different sets of data and incorporating medical devices with the Medical Internet of Things allows clinicians to work on and provide feedback, which extensively helps improve patients’s lifestyles, including home-based data attainment processes [9]. The advent of IoMT has enabled the availability of diverse data sets, which in turn facilitates continuous observation and monitoring of patients’s lifestyle-related diseases over an extended period of time. This advancement also empowers the analysis and identification of additional evidence, for instance, ambient conditions, comprehensive diagnostics, and therapeutic alternatives. Particularly in sectors such as dementia, where the characteristics of various stimuli need to be scrutinised to comprehend their attributes and their evolution over time. However, various risks may result due to the conciliation of devices, data infringements, and privacy or policy breaches. Moreover, the upgrade of the device and software configurations or modifications in the system that affect data analysis could lead to increased difficulty and potential risks [10].

The attention towards IoMT applications has notably grown, particularly in the past 5 years. Concurrently, sensors and medical devices have made significant advancements, incorporating crucial features. Consequently, a plethora of solutions can now be provided to address various critical conditions. The research work carried out in this particular area involves the development of prototypes, the exploration of new platforms for processing and visualising medical data, the creation of innovative architectures, interoperability, the enhancement of security measures, and so on. As there are plenty of sensors and devices available on the market, a scenario in an Intensive Care Unit (ICU) has been considered. Moreover, policies and guidelines have been designed for installing IoT technology on healthcare devices. Hence, an extensive understanding of contemporary deployments and technology in the healthcare field is considered to be quite fruitful for future research and the further advancement of Medical IoT devices. Therefore, the research put emphasis on the recent technology of the IoT in the healthcare sector, the IoT platforms for managing and processing medical data, and the various use cases of the IoT devices applied in the medical sector.

## 2. Literature Search and Article Selection

In order to identify the potential research spaces on the medical applications of the Internet of Things, a definite guideline offered by Kitchenham and Charters (2007) [11] and Page et al. (2021) [12] has been espoused to pinpoint the pertinent articles for this systematic review. The purpose of this search approach is to identify the suitable articles required to carry out this review [13]. To find and synopsise the results, various peer-reviewed documents, reports, websites, and different sources have been studied.

Now, one of the most vital tasks for this widespread searching is to use suitable search strings. Table 1 demonstrates the keywords to identify and summarise for this systematic review, which were carefully chosen on a trial-and-error basis.

Then, an amalgamated output of the individual searches generated 986 articles and related documents, which are after the year 2001, and duplicates have been removed for this study. After that, the selected articles have been checked using the inclusion–exclusion criteria as shown in Table 2.

**Figure 1:**
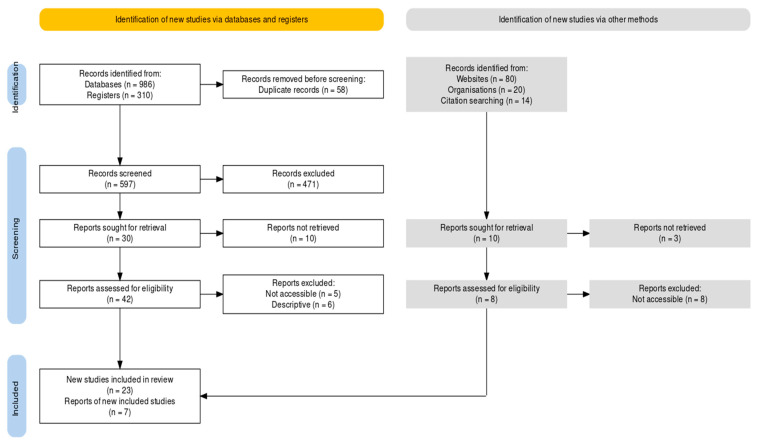
The PRISMA approach for systematic deduction of IoMT perspectives.

Now, after removing all the duplicates and using the inclusion–exclusion standards, a total of 597 unique records have been identified. By examining the abstracts and titles of the articles and excluding the pilot studies and documents less than 2001, a total of 42 eligible records have been obtained. Moreover, various details from websites, organisations, and citation searching have also been included in this study, and finally, 23 new studies have been added to the review. It is to be noted that unobtainable records and studies containing only discussion without further details have been excluded. Moreover, it can be mentioned that this review is distinctive, especially in terms of IoMT sensor circuitry details, as generally all the articles focus on IoMT and its data management process. Figure 1 exhibits the PRISMA approach [14] for this systematic review.

## 3. Internet of Medical Things (IoMT)

On the Internet of Medical Things (IoMT), a lot of sensors and different medical gadgets are utilised to collect data from patients and then to assess and provide suitable medications and suggestions to the patient. In order to primarily select the suitable sensors for this review, an Intensive Care Unit (ICU) scenario has been considered. The most commonly used sensors and devices in any ICU in general have been chosen, and the details are provided below:

### 3.1. IoMT Sensor Monitoring Circuits

The Medical Internet of Things (IoMT) is a subset of the Internet of Things that offers enormous potential for the healthcare sector. This sector’s products and services advance healthcare, ease the burden on doctors and nurses, and enable patients to receive care outside of hospitals or at home. The IoMT application’s framework makes it easier to combine the benefits of cloud computing and IoT technology with the medical industry. Additionally, it defines the methods for transmitting patient information from multiple sensors and medical devices to a particular medical service-providing network. The configuration of various IoT medical system/network components that are logically coupled in a medical context is the topology of an IoMT. It uses a variety of sensors, including sphygmomanometers (blood pressure monitors), thermometers, endoscopy monitors, pulse oximeters, EEGs, ECGs, EMGs, and so on, to read patient conditions at any given time [15].

Here are a few examples of IoMT-enabled sensors, along with their circuit diagrams, that will boost the connected medical field:

#### 3.1.1. Glucometer Monitoring

**Figure 2:**
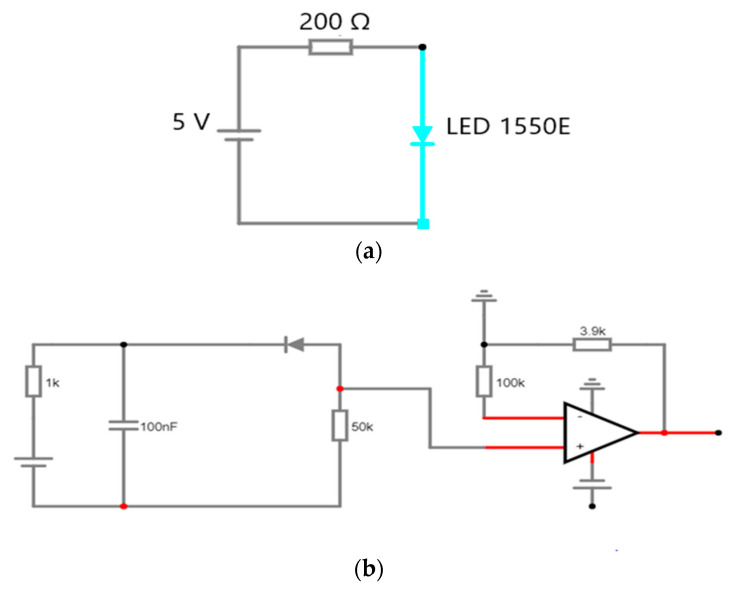
Glucose sensor: (**a**) transmitter circuit and (**b**) receiver circuit [17].

The device captures the glucose information in the blood of a human. A drop of blood from the human body is positioned on a one-time test strip, which is read by the glucometer to determine the glucose level in the blood. Figure 2 demonstrates the glucose level monitoring circuits [16].

Subsequently, diabetes is a health condition where the body’s blood sugar or glucose levels remain elevated for a longer period of time. It is considered one of the most widespread illnesses among people. Type I diabetes, type 2 diabetes, and gestational diabetes are the three main forms of diabetes that are typically present. Three tests—the random plasma glucose test, the fasting plasma glucose test, and the oral glucose tolerance test—can be used to identify the disease and its many kinds. However, “fingerpicking”, followed by the measurement of blood glucose levels, is the diagnostic technique that is most frequently used to identify diabetes. Numerous wearable devices for blood glucose screening that are non-invasive, cosy, practical, and secure have been created using recent advancements in IoT technology [18,19,20,21]. For real-time blood glucose level monitoring, an m-IoT-based non-invasive glucometer has been proposed in ref. [22]. In this case, an IPv6 connection was applied to connect the wearable sensors with healthcare providers.

#### 3.1.2. Temperature Sensor Monitoring

**Figure 3:**
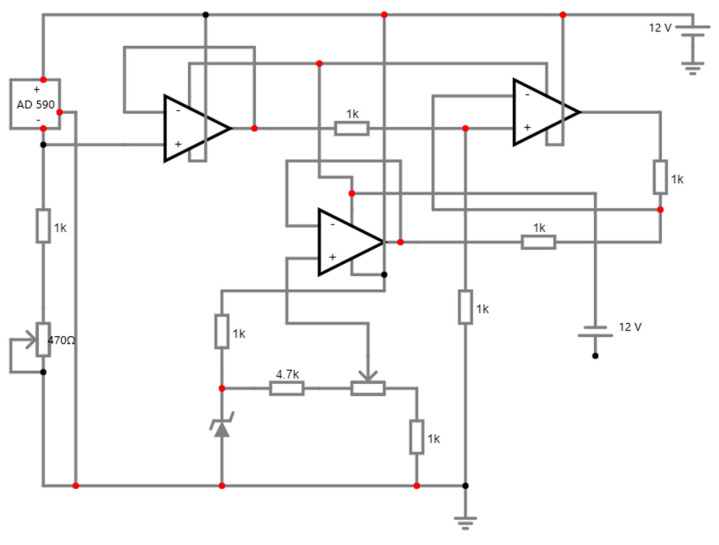
Temperature sensor circuit [23].

This sensor offers patients the ability to determine the body temperature, and it is one of the most important sensors as a lot of diseases change their characteristics, which can be determined by monitoring the temperature, and also some disease conditions can be checked by evaluating the body temperature. Then the doctor or the physician can determine the treatment based on the conditions. Figure 3 contains the temperature sensor circuit diagram [16].

An essential component of many diagnostic procedures, human body temperature serves as a sign of the preservation of homeostasis. Additionally, certain conditions, including trauma, sepsis, and others, can cause a change in physical temperature. The doctors can conclude about the patient’s health status in numerous disorders by keeping record of the patient’s temperature fluctuation from time to time. Operating a temperature thermometer that is either fastened to the mouth, ear, or rectum is the traditional method of taking temperature readings. The problem with these treatments is that they leave patients with little comfort and a significant risk of infection. However, a number of solutions to this issue have been put forward in light of current developments in IoT-based technologies. An ear-mounted, 3D-printed wearable device that utilises an infrared sensor to monitor the core physical temperature of the tympanic membrane was proposed in [24]. A wireless sensor module and an information-processing unit were built into the device. Here, the surroundings and other physical activity have no impact on the temperature that is being monitored.

#### 3.1.3. Blood Pressure Sensor Monitoring

**Figure 4:**
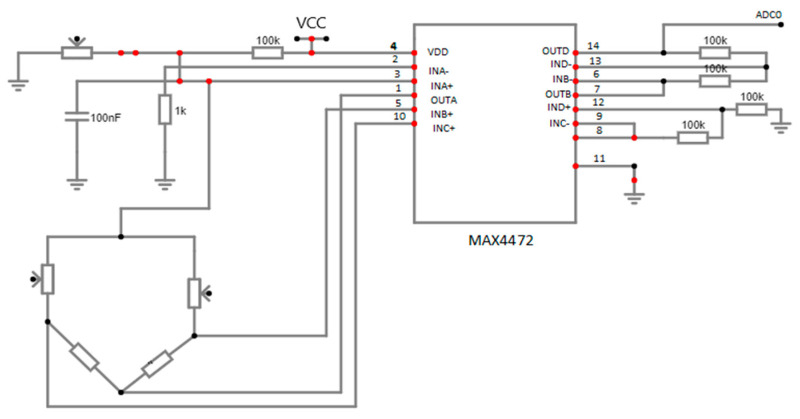
Blood pressure sensor circuit [26].

The blood pressure sensor captures the numbers for two different states, i.e., systolic and diastolic blood pressures. It allows the doctor to monitor the cardiac conditions of the patients [16,25]. Figure 4 illustrates the blood pressure sensor circuit.

The pressure sensor used in this circuit is primarily intended to measure blood pressure. It uses a ceramic chip and nylon plastic for high linearity, little noise, and low exterior stress. Moreover, it applies the internal standard method and the temperature compensation method to increase precision, steadiness, and repeatability [26]. The evaluation of blood pressure is a compulsory step in any diagnostic process (BP). The most usual method of taking blood pressure calls for at least one person to record the data. However, the method by which BP was previously monitored has changed as an outcome of the combination of IoT and other sensing technology. For instance, in [27], a wearable cuffless gadget that can gauge both systolic and diastolic pressure was suggested. The information that was captured can be stored on the cloud. Additionally, the effectiveness of this gadget was evaluated on 60 people, and its accuracy was assured.

In addition, researchers have recently put forward a non-invasive and wearable multi-biosensor for incessant and prolonged monitoring of BP while exercising for different medical tests. Three pulse transit time (PTT)-BP assessment models have been assessed, and results demonstrate that the impedance plethysmography waveform and arterial impedance provide superiority compared to others. This newly designed blood pressure monitoring system has been developed to be connected to the cloud to organise and maintain health data using IoT [28].

#### 3.1.4. Airflow Sensor Monitoring

**Figure 5:**
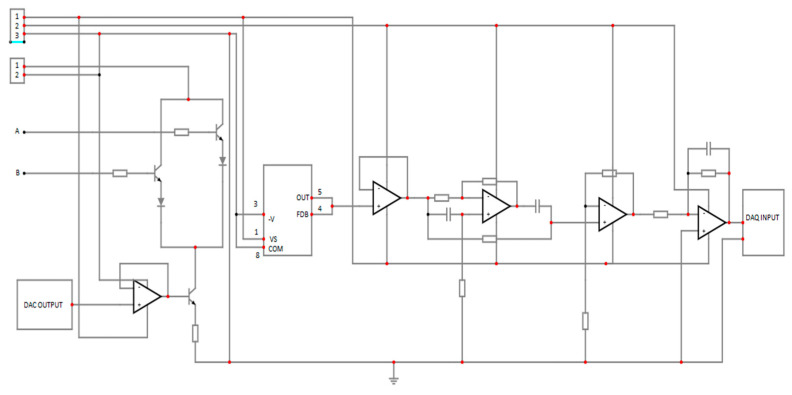
Oxygen saturation sensor circuit [29].

This sensor allows monitoring the breathing rate of the patient who requires respiratory support, which helps the physician determine the disease type and conditions, and therefore medicines can be prescribed accordingly. Figure 5 shows the oxygen saturation circuit diagram [16].

An important parameter in healthcare analysis is pulse oximetry, which is a non-invasive way of contemplating oxygen saturation. Real-time monitoring is offered by the non-invasive technique, which also eliminates the drawbacks of the traditional method. The incorporation of IoT-based technologies has improved pulse oximetry, which has substantial applications in the healthcare sector. A non-invasive tissue oximeter, which assesses the blood oxygen saturation level, heart rate, and pulse characteristics, was proposed in [27,30]. Additionally, by utilising different connection technologies like Zigbee or Wi-Fi, the captured data might be sent to the server. A decision for medical intervention was made in light of the recorded data. An alarm strategy that notifies patients when the oxygen saturation attains a disparaging level was observed in another study [30]. Moreover, to facilitate the continuous and longitudinal monitoring of diverse physiological signals, including body temperature, blood pressure, and electrocardiography, the researchers have introduced a wireless and malleable biosensor patch. This flexible patch design has been optimised to possess exceptional mechanical stretchability and adaptability, ensuring a dependable and enduring addition to the arched skin surface. Additionally, a setup consisting of an IoMT platform, cloud server, mobile application, and remote monitoring using a website was illustrated for health management and diagnosis [31].

#### 3.1.5. ECG Sensor Monitoring

The electrocardiogram (ECG/EKG) sensor is a diagnostic apparatus that is frequently utilised to determine the electrical and muscular functionalities of the cardiac system. ECG/EKG is one of the most important medical check-ups, and it assists in assessing numerous cardiac tests, for instance, myocardial ischemia, infarction, heart failure, arrhythmia, and so on [16].

**Figure 6:**
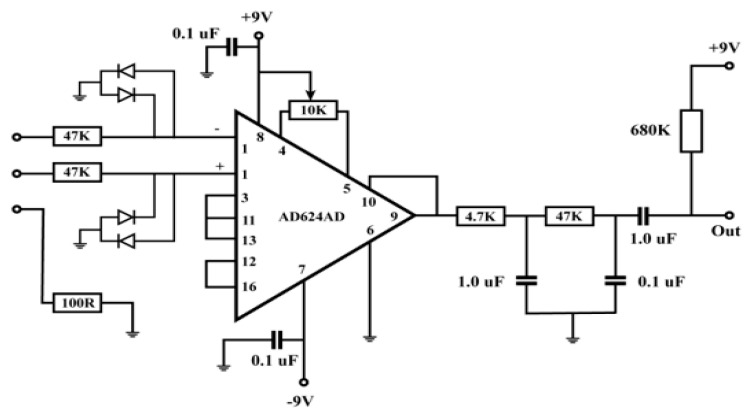
Circuit diagram of an ECG sensor [32].

From the Figure 6, the amplifier receives inputs from the electrodes, which are generally connected to the body. As the signals are quite small and the amplifier can be prone to different noises, it is imperative that the cables attach the electrodes to the circuit input [32]. Also, an electrocardiogram (ECG) depicts the electrical activity of the heart as a result of the atria and ventricles’ depolarization and repolarization. An ECG serves as a tool for diagnosing various cardiac disorders and offers information on the fundamental rhythms of the heart muscles. Arrhythmia, a prolonged QT interval, myocardial ischemia, etc. are a few of these problems. Through ECG monitoring, IoT technology has initiated potential utility in the primary identification of heart issues. IoT has been used to monitor ECG in numerous studies in the past [25,33,34,35,36,37,38]. A wireless information assemblage system and a receiving processor are the two components of the IoT-based ECG monitoring system proposed in the study reported in ref. [25,38]. A search automation system has been applied to quickly find cardiac irregularities. A compact, low-power ECG monitoring system that was unified with a t-shirt was proposed in [39]. It accumulated high-quality ECG data by applying a biopotential chip. After that, Bluetooth was used to send the recorded data to the end consumers. A mobile app could be used to visualise the recorded ECG data. A minimum of 5.2 mW of power might be used to run the suggested system. After merging it with big data analytics to administrate increased data stowage capacity, real-time screening in an IoT system may be achievable.

#### 3.1.6. EMG Sensor Monitoring

An electromyography (EMG) sensor is used to determine the electrical activity of muscles while inactive and at the time of contraction. EMG signals have been used in different clinical and biomedical fields. It is primarily used to diagnose neuromuscular diseases, lower back soreness, kinesiology, and disorders of motor control. EMG signals are also used to manoeuvre prosthetic devices, for example, prosthetic hands, arms, lower limbs, and others [16].

**Figure 7:**
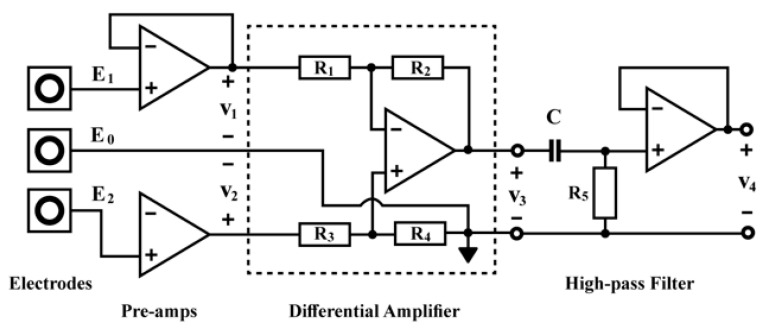
Circuit diagram of EMG sensor monitoring [40].

From Figure 7, two 9 V power supplies have been connected, including the electrodes attached to the muscles measuring the voltages E1 and E2, entering the preamps around three inches apart from the top and lower ends of the biceps, and the reference electrode on top of the elbow. The output is then attached to the oscilloscope to check the results. While the capacitor impedes the DC voltage, the fourth op-amp operates as the high-pass filter in between the differential amplifier and the oscilloscope probe. The output can then be observed and assessed [40].

#### 3.1.7. Breathing Rate Sensor Monitoring

Persistent illness like asthma compromises the air passages and leads to breathing issues. Asthma can cause the air passages to taper as a result of the airway oedema. This is a result of numerous medical conditions, such as wheezing, coughing, chest pain, and shortness of breath. An asthma attack can be triggered at any time, and the only thing that can save the patient at that point is an inhaler or nebuliser. As a result, real-time observation of this situation may be necessary. In recent years, many IoT-based systems for monitoring asthma have been developed [41,42,43]. A discrete sensor was utilised to observe respiratory rate as part of a discrete IoT solution for asthma sufferers that was prescribed in [44]. Caretakers have access to the health information saved on a cloud server for monitoring and diagnostic purposes. An LM35 temperature sensor is used by Raji to detect the breathing rate in his proposed respiratory monitoring and alert system [45]. The air that was inhaled and exhaled was measured to achieve this. The health centre received the respiration data, which was then shown on a web server there. Once a threshold value was obtained, the proposed system also sounded an alarm and immediately informed the patient.

**Figure 8:**
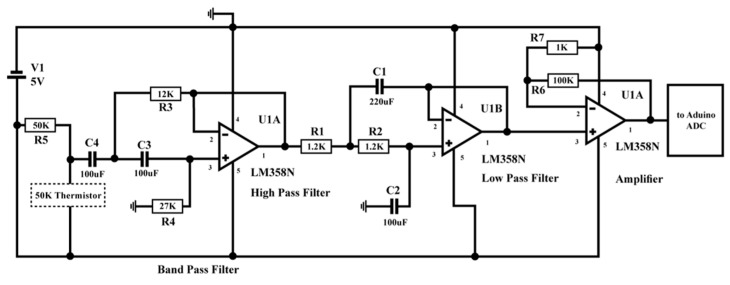
Circuit diagram of a breathing rate monitoring sensor [46].

The breathing rate monitoring circuit, as demonstrated in Figure 8, contains a 50 K negative temperature coefficient thermistor attached to the nebuliser mask. Thermistor resistance lessens during breathing-out time because of hot air, and vice versa during breathing-in time. A change in the resistance value is then identified as an ac signal, which is passed through a band-pass filter (low cut-off = 0.10884 Hz and high cut-off = 0.8942 Hz) so that the dc and high-frequency noise are removed [46].

#### 3.1.8. Mood Monitoring

Mood tracking is operated to conserve a healthy psychological state and give important information about a person’s emotional state. Additionally, it helps medical experts treat a variety of mental illnesses like depression, stress, bipolar disorder, and more. Human interpretation of their mental state is improved by individual monitoring of their psychological state. According to [47], a CNN network is used to analyse and classify an individual’s mood into six different categories: joyfulness, thrilled, sad, calm, distressed, and anger. An interactive technology called “Meezaj” was used in a related study [48] to measure real-time mood. The software also demonstrated the magnitude of happiness in decision-making and helped policymakers discover the critical elements that define an individual’s joyfulness. With the incorporation of a cutting-edge machine learning structure, anxiety may now be interpreted beforehand using heart rate. The system can also talk to the patient about their stress level [49].

**Figure 9:**
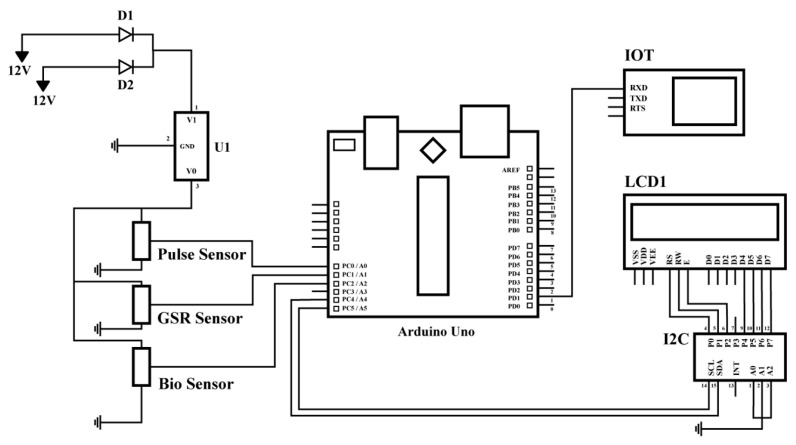
Circuit diagram of a breathing rate monitoring sensor [50].

From Figure 9, the GSR (Galvanic Skin Response) sensor identifies the variations in skin temperature; the abnormal cardiac rate is distinguished by the pulse sensor; and the nanobiosensors monitor the blood pressure, seratonine, and other relevant parameters. The data from all these components is accumulated using Arduino Uno, which is then passed onto the IoT cloud for analysis and storage. As the data can be found in real time, nurses or any medical personnel can receive a warning if depression is observed [50].

### 3.2. IoMT Device Applications

Various applications of IoMT devices have been briefly discussed in the following, and the information has been enumerated in a thematic group to obtain perceptive outcomes.

#### 3.2.1. Monitoring Devices

##### MySignal

MySignal is a tenet for creating healthcare gadgets and eHealth software that is used to create new medical devices by adding sensors or even creating eHealth web apps. Over 20 biometric parameters, incorporating blood pressure, oxygen levels in the blood, muscle electromyography signals, glucose levels, galvanic skin response, lung capacity, snore waves, patient position, airflow, and body scale parameters, can be measured using MySignals (weight, bone mass, body fat, muscle mass, body water, visceral fat, basal metabolic rate, snore, and body mass index). MySignals is the most all-inclusive eHealth platform on the market, thanks to its extensive sensing portfolio [15]. Moreover, once a suitable sensor has been chosen to assess the data, it then conveys the information to the MySignals Web Server Mode [51].

##### QardioCore

QardioCore is an ECG monitor intended to deliver continuous, high-quality healthcare information. Wearers can use these gadgets as part of their regular routine at work, the gym, or when out and about. According to the statistics, people can more effectively monitor medical issues, including excessive cholesterol and blood pressure. Without requiring actual visits, it also transmits data to medical facilities that keep tabs on ailments including diabetes, heart problems, and weight gain [52]. Moreover, QardioCore ECG monitoring is a prescription-based device with Bluetooth and IoT capabilities that only functions using prescriptions. Once the report has been received, it is then assessed by medical personnel and advised accordingly [53].

##### Smart Thermometer

The Kinsa smart thermometer serves three main functions: diagnosing patient sickness, providing analysis for finer care, and mapping human illness through gathering information. Many American homes already have versions of Smart Ear and Sesame Street [52].

##### UroSense

For patients undergoing catheterization, UroSense is a catheter with a transmitter that tracks urine flows and basal body temperature. The early detection of an infection’s symptoms can lead to better treatment and prevention strategies, thanks to the careful monitoring of these two factors. In order to help manage illnesses, the gadget also suggests conditions like diabetes or prostate cancer and relays this information to medical healthcare service providers [52].

#### 3.2.2. Tracking and Tracing

##### Zanthion

A Zanithon is a healthcare alert device held by a patient as a piece of jewellery or clothing. It fuels a network of linked sensors that track the wearer’s wellbeing and health. An alarm is issued to relatives or friends who can assist if a patient falls out of bed or is immobile for an extended period of time [52]. Also included are a cardiac rate monitoring facility, real-time GPS for milliseconds response, and if the body temperature is not in range, mainly in the flu season [54].

##### UP by Jawbone

A unique physical fitness tracer is available from Jawbone. Instead of merely totalling calories and steps, it may be used to keep track of all healthcare aspects, including weight, sleep patterns, activity levels, and food. This information can then be used to help the user make well-informed health decisions. Even outside of the actual medical institution, some patient advocacy organisations are using it to help people who are struggling with their weight and general health [52].

##### NHS Test Beds

NHS test beds are intelligent, networked beds used in the UK’s NHS system that track data and monitor patients. They save money and time by coalescing wearable monitors with other data sensor sources, making it possible for older patients and people with chronic illnesses to track their advancements and problems further effectively and efficiently [52].

##### AwarePoint

IoMT sensors are placed by systems like AwarePoint to monitor every detail of the caretaking process. In what they refer to as “location-as-a-service,” this offers location tracing on patients and healthcare equipment. The system’s goals include enhancing employee and patient happiness, streamlining asset management, and improving patient flow [52].

##### Apple Watch

To assist patients with severe depressive illnesses, Takeda is evaluating the use of Apple Watch software. In addition to allowing tracking of moods outside of medical sessions, the app will look for symptoms [52].

#### 3.2.3. Device Sensors

##### Swallowable Sensors

A swallowable sensor allows patients to keep away from colonoscopies by ingesting a sensor the size of a cod liver oil tablet. Instead of more intrusive surgeries, this sensor can diagnose issues related to illnesses, including irritable bowel syndrome and colon cancer [52]. Recently, Hou et al. (2023) analysed and calculated that for the radiation dose, the swallowable dosimeters are five times more precise compared to the standard methods of dosage selection [55].

##### Propeller’s Breezhaler Device

Propeller’s Breezhaler Device is a networked sensor that facilitates the administration of COPD or asthma. Every time the pump is used, the sensor, which is fastened to the top of the device, collects data. With the use of a mobile app, the user may do everything from gather information on triggers to allow family members and doctors to control use. It is claimed to enhance the number of days without symptoms and decrease the number of asthma attacks [52].

##### Non-Invasive Sensors, Microchips, and Other Miniaturised Electronics

In 2014, Google stated that Novartis would help it develop a smart, connected contact lens. The contact lens would be embedded with “non-invasive sensors, microchips, and other miniature electronics”. For those who cannot read without glasses, they can monitor medical disorders like diabetes and help repair vision problems to re-establish the eye’s natural autofocus [52]. Moreover, flexible miniaturised sensors (FMS) for physiological monitoring are quite noteworthy due to their various applications in gathering health-related information, assessing, and managing the wellbeing of patients [56].

#### 3.2.4. Device Management

##### ScreenCloud

ScreenCloud is already in use by healthcare service providers and medical professionals, with applications that go far beyond conventional digital signs. Consider the group that uses digital signs and video art to enhance patient welfare in hospitals, with impacts such as reduced tension and anxiety in patient waiting areas that can be seen [52].

##### Medication Dispensing Service

Patients who might have trouble managing their medications on their own are the main focus of the Medication Dispensing Service and other intelligent pill-dispensing gadgets. MDS dispensers alert patients when it is time to refill their medications by pre-filling them with the necessary dosage on a certain day. The information can be traced and given to the patient’s health-care service provider if a medication is missed [52].

##### Medication Supervision

In the medical sector, medication adherence is an extensive issue. Patients’ unpleasant health consequences may become more severe if their pharmaceutical regimen is not followed. Elderly people are more likely to have medication non-adherence because, as they grow older, they develop critical diseases like dementia and comprehensible loss. Therefore, it is challenging and difficult for them to strictly adhere to healthcare service providers’s orders. The use of IoT to track patient medication compliance has been the subject of substantial research in the past [49,57,58,59,60]. A discrete medical box that can remind people to take their prescription was created in [61]. Each of the three salvers in the box holds medication for three different dosages (morning, afternoon, and evening). The system also takes several crucial health measurements (blood sugar level, blood oxygen level, temperature, ECG, and so on). The cloud server is then delivered with all of the recorded information. The two end users were able to communicate thanks to the smartphone app. Health care service providers and patients can use the cellular app to access the recorded data.

##### Wheelchair Management

For people with deficient mobility, a wheelchair is an essential component of everyday life. It offers both psychological and somatic assistance. The use of a wheelchair is, however, constrained when the condition is brought on by brain damage. The current research focuses on coupling these wheelchairs with the navigation and tracking system. IoT-based technologies are currently representing possible outcomes for reaching this objective [62,63,64,65,66]. A real-time obstacle avoidance system and an IoT-based steering system have been presented in [67]. By applying image-processing algorithms to the real-time films, the steering system can recognise obstructions. Wheelchair management has become easier and more participatory for patients, thanks to the usage of mobile computers. A smart wheelchair was created by fusing multiple sensors, mobile technology, and cloud computing [68]. A mobile app that is part of the system enables patients to communicate with their wheelchairs and carers. The software also makes it possible for caretakers to keep an eye on the wheelchair remotely.

##### Rehabilitation System

A patient with a disability can regain their functional abilities with the help of physical medicine and rehabilitation. Recognising the issue and assisting patients in returning to regular life constitutes rehabilitation. IoT is used in rehabilitation in a variety of ways, including the treatment of physical limitations including cancer, sports injuries, and stroke [69,70,71,72]. Using a multimodal sensor that traces the patient’s gait pattern and assesses motion parameters, a discrete walker reformation system has been proposed in [73]. The smart walker recorded several motion matrices, such as orientation angle, elevation, force, and so forth, while a patient used it. The doctors gauged this information and originated diagnostic findings utilising a cellular phone app. Additionally, a robotic hand, a discrete wearable wristband, and a machine-learning algorithm were combined to create a stroke rehabilitation device [74]. A low-power IoT-based textile electrode that monitors, pre-processes, and transmits the biopotential signal was used in creating the armband.

#### 3.2.5. Other Notable Applications

The utilisation of IoT is varied and not just restricted to the aforementioned purposes. The number of IoT applications is quickly advancing along with the speedy development of technology. A few research fields that previously did not precisely encapsulate the integration of IoT gadgets are now efficiently utilising this technology. This could involve the treatment of cancer, distant surgery, the identification of haemoglobin, aberrant cellular growth, etc. A brand-new IoT-based structure for cancer treatment was put forward in [75], and it included chemotherapy and radiotherapy as well as different stages of cancer treatment. The doctors provided online consultations through a mobile app. The healthcare professional may consult the patient’s lab test results that were kept on a cloud server to determine the best timing and amount to provide medication. Lung cancer detection utilising various cutting-edge machine learning algorithms and an IoT-based system is another possible use [76,77,78].

Additionally, a recent study made the case for employing an IoT-based system to identify skin lesions [79]. Also, a novel approach to low-level laser therapy (LLLT) for automated facial dermatological ailment diagnosis was proposed by researchers, which involves the integration of a deep neural network and MIoT platform [80]. Moreover, a wireless and flexible light-emitting diode (LED) patch integrated with the MIoT platform has been developed for injury remedial applications. This cutting-edge patch is characterised by its high-efficiency thermal steadiness, device homogeneity, and mechanical sturdiness, making it suitable for skin-attachable phototherapies and clinical uses [81].

Moreover, a state-of-the-art LED therapy system that employs deep learning techniques for the automatic identification of facial acne vulgaris has been proposed. Additionally, a healthcare IoT (H-IoT) platform has been proposed to establish connections between smartphone apps, a cloud server, and the LED therapy device [82]. The next-generation surgical training framework was created by Cecil et al. using IoT [83]. The tool created a teaching environment through virtual reality and offered a platform for surgeons from various locations to communicate with one another. A collaborative human-robot system that can successfully carry out minimally invasive surgery has been proposed in [84]. The amount of haemoglobin in the blood can be checked with portable equipment [85]. In order to measure haemoglobin, the gadget used photoplethysmography (PPG) sensors, a light-emitting diode (LED), and photodiodes. By contrasting the outcomes with the results of the recognised colorimetric test, the effectiveness of the gadget was further demonstrated.

### 3.3. How IoMT Works and Its Data Storage

**Figure 10:**
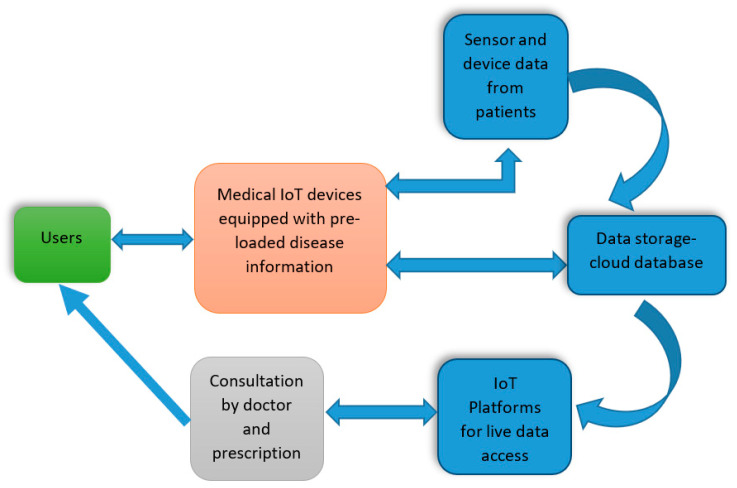
Circuit diagram of a breathing rate monitoring sensor [16].

IoT in medical fields such as remote medical observation, physical fitness curriculums, immedicable diseases, and elderly care and monitoring can significantly improve patients’s experiences. Hence, different healthcare gadgets, sensors, diagnostic, and imaging devices are now considered smart devices, with the core section being IoT, and IoT-related medical devices are anticipated to be cheaper, provide a much higher quality of life, and provide an easier and more flexible user experience. In the case of healthcare providers, IoT can determine the time required for supplies and also help minimise device downtime using remote delivery [86]. Therefore, in general, IoT-based medical devices will capture patient data from different sensors, match it with the existing symptoms or the data inserted to validate the diseases, and then store it in the cloud and in different databases. The doctor can then take a look and authorise medicines or further testing as required. The following diagram (Figure 10) demonstrates a simplified way of understanding the working procedures of Medical IoT [16]:

Users: The user will enter their personal details, which will be stored in the system along with their disease symptoms.

IoT Devices: The Medical IoT devices will capture and match the patient data with the pre-loaded disease symptoms and process further to analyse the details of the disease and a suitable cure for the patients. However, if the disease symptoms and details are not found in the system, then the system contacts the doctor, and the doctor will discuss the details with the patient (user), diagnose the disease, and perform tests. Once the doctor has more information on the disease, the results and related data are uploaded to the system. Information about the patients and their diseases is deposited in the Cloud and database systems. When the output matches the disease information, then the system produces the required prescription, consisting of general medicines for the defined disease.

Sensors: In order to perform different tests for the patients to determine the diseases, there are numerous types of sensors that can be implanted in Medical IoT devices, for instance, glucometers, temperature sensors, ECG/EMG sensors, and others.

Data Storage-Cloud Database: As the central IoT device will store all the patient information in the cloud-based database system, it can therefore perform future analysis for others. The database will store patient’s details, disease symptoms and diagnosis results, information on the tests performed along with the reports and results, prescriptions and medicines, doctor or physician consultation details, sensor, and device information on the location of log-in and device monitoring on the cloud.

IoT Platforms: From the cloud, doctors and medical personnel can easily access detailed information about the patient and also live data coming directly from various sensors and gadgets.

Consultation and Prescription: As per the input provided by the patient and also based on the diagnosis details of the disease, the IoT device will try to match suitable prescription information in the pre-loaded prescription file, including proper medication, and notify the user. Moreover, the user can also check the details and query if there is anything that needs to be clarified.

Subsequently, Medical IoT devices can also be connected using wireless re-programmable devices and sensors consisting of healthcare software applications or apps installed on cellular devices. An IoMT system is generally considered the healthcare system, which includes observing and assessing medical devices. These devices are able to track a patient’s health remotely and put it back in the database. The back-end system then inspects the received data and provides warnings to the doctors. Hence, the doctor can assess the warnings and prepare for any emergencies as necessary. In general terms, the data extracted by the monitoring devices (such as smart watches, cell phones, etc.) seems to be quite complex and is usually construed against the medical data of the patients. Therefore, this structure can be utilised in household care units, clinics, and other places. The IoMT structure generally portrays a complex ecosystem that consists of different elements and arrangements such as healthcare gadgets, smart devices, hubs, gateways, Cloud services, databases, big data, clinical information systems, etc. However, similar to any new technology, IoMT also faces various challenges, for instance, interoperability, functionalities, device constraints, and security [10]. Advanced medications and processes using IoMT would certainly elevate patients’s quality of life.

In recent times, physicians have also relied a great deal on smart medical devices as their primary method of detecting diseases. These medical devices, from a bigger standpoint, have minimised the workload of the medical service providers by observing the patient’s physical condition intensively and proceeding with pragmatic steps if there is any significant change in the patient’s data [16,86,87].

Subsequently, technological innovation in fitness devices has also attracted a huge number of customers seeking heart rate monitoring, temperature reading, calorie consumption, burn rate, etc. Since the devices enabling these features are very user-friendly, it is expected that market demand will go up globally in the coming years. In terms of products, the global market is divided mainly into three categories, i.e., therapeutic gadgets, diagnostic and observing devices, and injury prevention and reformation devices. Amongst them, the diagnostic and observing device is considered to be controlling the international market. Features such as easy access to fitness details, reliable health data measurements for instance, diabetes, blood pressure, etc., and accessibility of these devices in different ranges of cost help this segment to be in the lead. Also, as people become more aware of their fitness, the diagnostic and observing device section is therefore expected to manipulate the market. IoT-oriented medical systems are key to the substantial growth of ‘connected’ medical systems. Features such as tracking, tracing, and monitoring patients are vital for the connected health care system. However, reliance on the health care system using IoT is on the rise, as the modern world requires enhanced quality of medication and care while at the same time reducing cost. For general queries, IoT in fact abolishes the requirement for a doctor as the system can monitor patient data using different sensors and a cloud-based complex system to evaluate the accumulated information and transfer the results wirelessly to healthcare professionals for further investigation if required [87].

Capturing the health data of the patients is very important, regardless of the data generated from the electrocardiograms (ECG/EKG), fatal monitors, blood sugar levels, or temperature monitors. These data would call for investigations by doctors or healthcare professionals, which creates an opening for discrete electronic devices to produce more essential data and reduce the requirements for more uninterrupted doctor-patient interactions. One of the recent applications of IoMT is the installation of smart beds in hospitals, which allows the nurses to monitor if the patient is struggling to get up and also when the bed is in use. These beds are also able to axiomatically regulate the proper force and reinforce it for the patient without any physical involvement [87]. With the current inclination towards a connected healthcare system and extensive use of healthcare technology, the idea of developing a ‘Smart Hospital’ would become an actuality by 2020 [88]. Therefore, companies focusing on healthcare technology tend to invest largely in the IoT.

Currently, the majority of electronic devices offer some sort of connectivity, ranging from biosensors to X-ray machines enabling Wi-Fi or Bluetooth. Another prime example of the IoT-based system is the Chic Fridge by Weka for vaccines, where the primary aim is to sort out the vaccine management risks and issues, for example, keeping the vaccines at an indorsed temperature, consistency of electricity, and inventory mismanagement. The Weka Fridge provides a distant observation structure to safeguard that the vaccines are kept at the proper temperature, and a self-operating log system can confirm the updated information on the stored vaccines. As per the request from the physicians, the smart fridge can offer the vial without interrupting other medicines. In addition to the simplified process of obtaining the vaccines, this fridge also allows the physicians to access the data to be processed and investigated to develop a better vaccine plan [8].

### 3.4. IoT Platforms

An IoT platform consists of a multi-layer technology that allows provisioning, managing, and automating the connected devices in the IoT space. It generally links the hardware to the cloud using different connectivity selections, enterprise-level security mechanisms, and extensive data processing capability. For developers, an IoT platform offers a set of established options that facilitate the integration of applications for connected devices, scalability, and compatibility [89]. The following block diagram can demonstrate how the IoT platform interacts with the hardware and the applications.

**Figure 11:**
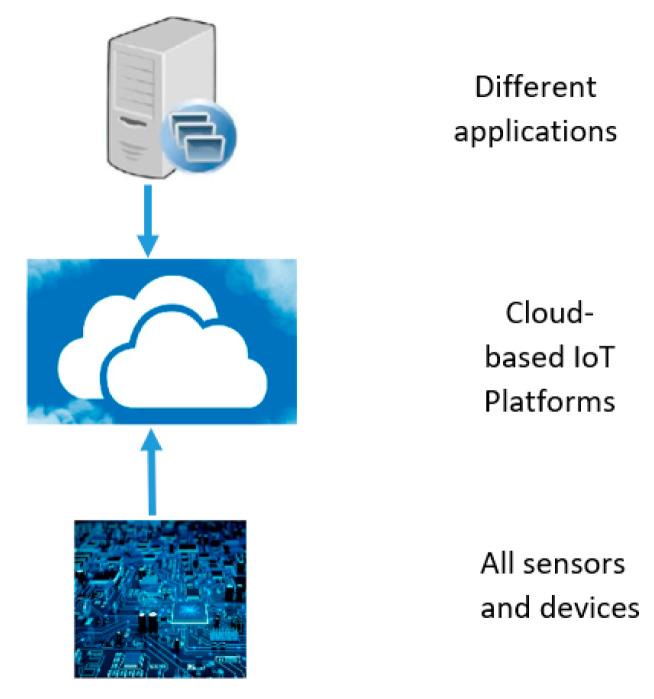
Basic block diagram of the IoT platform [89,90].

In Figure 11, IoT acts as a ‘middleware’, which interconnects the hardware and the various applications linked to it directly to the cloud. Hardware can include different sensors, devices, mobility, tags, beacons, health and fitness devices, consumer electronics, automotive, embedded hardware, and so on. Moreover, applications could consist of data storage and analytics, consumer applications, industrial applications, business applications, healthcare, and others. Data can be encompassed and analysed from this hardware, such as sensors and devices, as well as from various applications [89,90].

As the IoT platform functions as the central hub for connectivity, data extraction, and data generation, it is largely accountable for the connection between the hardware and the application layers. Also, the main responsibilities encompassed gathering data from various devices using diverse protocols and network topologies, remotely configuring and controlling devices, and managing devices and firmware updates on a regular basis. To be effectively applied in the real-time IoT ecosystem, this middleware is anticipated to generate seamless integration with a wide range of connected devices [90]. There are various IoT platforms available for different applications, but one of the decent IoT platforms in medical services is the Kinect HoloLens Assisted Rehabilitation Experience (KHARE). This tenet has been developed by Microsoft Enterprise Services alongside the National Institute for Insurance Against Accidents at Work (INAIL) to mimic the efferent neuron therapy capability. Using KHARE, the physicians can obtain access to the real-time data feeds, which allows them to assess and develop complete and adapted medications for patients irrespective of their physical locations. This platform links with Microsoft’s Azure IoT Suite, and using such features, healthcare professionals can access the information during a half-hour therapy period. This tenet has been undertaking a clinical investigation, which was supposed to be completed in January 2020. Moreover, IoT information analysis programmes, for instance, Kaa (KaaIoT Technologies, FL, USA), MindSphere (Siemens, TX, USA), and Azure (Microsoft, Washington, USA), permit information to be organised and accumulated from the respective IoT gadgets to conclude a significant result [8].

The hospitals in Singapore are also setting up digital platforms for the patients who have been cleared from the hospitals, through which they can offer patient care as well as access to different healthcare providers. This is to reduce the cost and the time required by the patients to visit the hospitals, as well as the follow-up visits. Due to the challenges in the healthcare system associated with cost, gaining access to health records, and enhanced health services with more features, there has been outstanding development in novel ideas and solutions. Hence, ‘smart’ healthcare systems provide upgraded services to the patients, offer more options to the medical teams, and also alleviate the business of healthcare systems [88].

Consequently, a number of health insurance companies are trying out digital platforms, including wearable devices and sensors focusing on wellbeing and acute disease management. Also, by providing incentives and rewards, the patients can be motivated to live healthy lifestyles. Hence, it would certainly lower the cost of supporting medical service personnel and medical service insurers. Moreover, this transformation in the digital healthcare system is going to draw attention in ‘health tourism’, with advanced features such as telemedicine for pre- and post-treatment check-ups, digital record management, and technological developments in healthcare such as precision medicine, limited surgery, and digital laboratory analysis. In the near future, countries with massive investments in the IoT space will move to the centre of the medical tourism market. Additionally, these digital medical records are processed and operated through digital transformation. Healthcare Information Systems (HIS) are slowly shifting from conventional paper-based records to electronic medical reports (EMRs), and this is one of the important advantages and drivers of the digital transformation. This offers up the seven-step process suggested by the Healthcare Information Management Systems Society (HIMSS) for hospitals to transform into ‘paperless hospitals’ [88].

Therefore, it can be understood from the following analysis that the IoT platforms can contain various information about the patients, where the medical doctors can retrieve the detailed history of the patients along with the latest updates and provide suitable suggestions. Users can also contact the medical personnel if there is an emergency or if they have any queries, which saves both time and money. In order to understand how Medical IoT devices work, a case study on COVID-19 has been considered.

### 3.5. Case Study of COVID-19 Assistance Using IoMT

The applications of Medical IoT devices in the health area offer medical centres and staff the opportunity to function more proficiently. Internet of Things (IoT) devices have become widely employed in a variety of applications in recent years, including discrete urban areas, factories, households, automation, and medical [91]. Sensors are used in these devices to acquire data about the physical environment. The world’s healthcare system is now overwhelmed as an outcome of the COVID-19 outbreak.

The COVID-19 epidemic sparked a slew of further forecasts. The Internet of Things is anticipated to play a vital role in the new normal scenario. This article offers, analyses, and suggests IoT-inspired applications in many industries during the epidemic, as well as difficulties and potential beyond the pandemic [92].

Numerous wearable, lightweight IoT devices [93] (p. 1) are available that might be utilised to minimise infectious diseases like COVID-19 and ameliorate medical solutions. Very few symptoms can be easily tracked by using IoT devices. If viral symptoms are identified, the gadget may inform both the user and the nearest health agency. This can improve medical intervention performance to identify, scrutinise, and rescue individuals in deprecatory conditions (e.g., the patient is failing to interact with the medicals at the appropriate time due to symptoms) and convey the information to other departments to find a rapid cure and keep civilians unharmed. It is considerably simpler to monitor patients from a considerable distance with the utilisation of technologies such as 4/5G and the cloud, especially for people who find it complicated to contact or access medical institutions.

The other pros of IoT gadgets in the medical sector are that they reduce human errors, which are more common than machine or AI faults. Taking into account that humans are imperfect, IoT can provide more accurate diagnosis and patient reporting.

The Internet of Things (IoT) technology plays a vital role in identifying the virus by using fever tests to consider some of the virus’s signs, restricting spread by imposing social distancing and supervising remote health observation, pollution and air quality management, occupancy control, and smart parking. Fever screening eliminates the need for human interaction and enables the identification of several targets. Sensors and cameras supply colour temperature scales and pictures. The first line of protection against the virus is pre-screening of personnel, disaster evacuees, or patients [94]. This does not ensure viral detection, but it can be used to assess whether the person has virus symptoms, and further inspections are performed for a definitive conclusion.

The SARS-CoV-2 virus causes the COVID-19 virus, which influences the respiratory system. In [95], researchers suggest a wearable strain sensor that detects the rate of breathing of the user’s and volume of the user’s respiratory system. Patients’ breathing conditions are being tracked and monitored by these sensors using the Internet of Medical Things (IoMT), and it gives constant updates to the clinicians engaged about the patient’s respiratory state. This also saves time since it allows the concerned health care service providers to make suitable suggestions remotely. The authors of [94,96] focus on a wearable IoT-based stress detection device. This is a suitable option for people experiencing worry, anxiety, and isolation as an outcome of the epidemic.

During the COVID-19 epidemic, contact tracing proved to be an effective way to secure personal information while keeping people well. Wearable light IoT devices, such as the Triax Proximity Trace, employ contact tracking to remove social distance by alerting users when they are not maintaining proper distances [97]. Enterprise departments can deploy wearable gadgets that enforce safety requirements without requiring cellular phones or downloads. Tags are transmitted via Bluetooth technology, which eliminates the urge to trace a user’s location. Filtered keys that alter every 15 min are also employed to secure users’ privacy. The Alberta government has released a COVID-19 contact tracing application. The software verifies positive instances and alerts Canadians who have been affected [98].

During a pandemic, medical personnel affiliated with ambulance services face more stressful and uncomfortable conditions. Ambulance IoT-assisted technology provides timesaving solutions by allowing specialists to advise employees on important procedures to cope with patients in emergency conditions [99]. IoT-based Emergency Medical Services (EMS) are excellent methods for saving lives in life-threatening circumstances. The authors of [100] suggest a structure that delivers real-time information on the number of accessible beds, all forms of blood levels, blood type availability, and doctor accessibility. During such enormous incidents with several victims, the ambulance can provide real-time data.

An IoT-based system has been formed to aid in the early-stage detection of coronavirus-infected individuals. To diagnose suspected coronavirus cases, faster region CNN with ResNet101 (FRCR) was used. The FRCR has a 98 percent accuracy rate [101]. For automated testing of COVID-19 from chest CT images, an attention-based deep 3D multiple instance learning (AD3D-MIL) method was developed [102]. The Bernoulli distribution of labels was used by AD3D-MIL for efficient learning.

The Internet of Things’ massive number of network devices that trace and alert to different kinds of illnesses assists in the development of a discreet network for medical service-provider management systems. Patient data are collected without the assistance of humans, which may be valuable in decision-making procedures.

Using the generative adversarial network, an auxiliary classifier model was created to generate synthetic chest X-ray pictures (GAN). CovidGAN [103] is the name given to the created model. COVID-19 was distinguished from other viral pneumonias using CovidGAN. CovidGAN was tested using 192 chest X-ray pictures. However, CovidGAN does not do cross-validation. Using ultrasound, X-ray, and CT scans, deep learning models were employed to identify COVID-19 suspicious cases [104]. VGG19 was used to create an automated categorization method. The pre-processing approach was utilised to reduce sample bias and improve image quality. Data fusion strategies, on the other hand, can improve classification accuracy. For the categorization of COVID-19 suspicious instances, a CNN-based transfer learning architecture was presented [105].

In this suggested system, eight pretrained CNN models were used: ResNet18, Inceptionv3, SqueezeNet, MobileNetv2, ResNet101, CheXNet, DenseNet201, and VGG19. This framework was evaluated using 423 COVID-19 pictures, 1485 viral pneumonia images, and 1579 normal chest X-ray images. To distinguish COVID-19 infection from other diseases, a 3D convolutional neural network (3DCNN) was created [106].

DCNN used online attention refinement and a dual-sampling approach. This network was utilised to isolate the infection locations and remove the uneven distribution of pneumonia-infected regions. DCNN was put to the test on 2796 CT scan pictures from 2057 patients. However, the precision of the affected area is still lacking [106]. To categorise coronavirus-infected people using chest X-rays, a deep learning-based chest radio classification (DL-CRC) system was suggested [105,106]. To create simulated coronavirus-infected X-ray pictures, DL-CRC employed a generative adversarial network and data augmentation. Four separate chest X-ray datasets were used to evaluate DL-CRC.

Medical IoT devices have greatly aided in the battle against the COVID-19 outbreak. Deep learning models based on IoT have been developed to lessen the tasks of healthcare personnel and doctors. However, IoT-based deep learning models, which do not take into account protective models against adversarial anxiety, are still susceptible to adversarial assaults [106,107].

The Internet of Things (IoT) employs a great number of networked gadgets to form a discrete network for medical service management. It monitors and notifies the patient of any form of ailment to assure the patient’s safety. It digitally records the patient’s information and data without requiring any human assistance. These data are also beneficial for making suitable decisions [100,108,109,110,111].

To put a halt to this pandemic, coronavirus-infected people must be diagnosed as soon as possible. IoT devices are employed to remotely retrieve information from COVID-19 patients for this purpose. These data are sent on to medical personnel in order for COVID-19 to be diagnosed [112]. These gadgets not only relieve the load on healthcare staff, but they also spot unexpected patterns in sensor data [105,113]. Using IoT-enabled devices, healthcare personnel can deliver better treatment for coronavirus-infected patients more quickly. There is a need to create an automated categorization system that makes use of the data offered by IoT gadgets. Many academics have recently used deep learning models to serve a variety of healthcare applications [9,15,25,33,34,35,36,37,38,52,103,114,115,116].

In light of the latest accomplishments of deep learning models for axiomated coronavirus detection, an IoT-based ensemble deep learning framework has been created. The suggested ensemble model will aid radiologists and medical personnel in classifying alleged patients as COVID-19 (+), pneumonia, TB, or healthy. Combining InceptionResNetV2, ResNet152V2, VGG16, and DenseNet201 creates a deep ensemble model [117]. The medical sensors can grab the chest X-ray modalities and use an ensemble deep transfer-learning model stored on a cloud server to identify the illness. For the experiment, a chest X-ray dataset with four classifications (COVID-19 (+), pneumonia, TB, or healthy) was employed.

According to the comparative study, the suggested model would assist radiologists in effectively and swiftly identifying suspicious COVID-19 patients. On 460 CT images, AD3D-MIL was trained and evaluated. Using CT scans, a multitask multiline deep learning system (M3 Lung-sys) was built for finding coronavirus-infected people [118,119,120].

Table 3 below outlines the COVID-19 assistance using IoMT devices, software, services, models, and networks, including features, pros, and cons:

## 4. Discussion and Challenges

The applications of Medical IoT devices in the health area offer medical centres and staff to function more proficiently, and patients also receive better treatment options. Using this technology, patients are getting everything they need at home without visiting the doctor every time.

However, the Medical IoT also faces a lot of challenges. For instance, there are a few devices that have been hacked, and keeping track of the total number of associated devices and the immeasurable amount of personal data the devices collect could be a substantial risk for cyber security. In 2017, the FDA took an exceptional decision to re-collect 450,000 pacemakers as these devices were found to be at risk of cyberattack. Johnson & Johnson also issued a warning to their customers regarding a security malfunction in one of the insulin pumps. An investigation by Synopsys found that only 51% of device manufacturers and 44% of healthcare companies stick to the FDA guidelines to lessen the security risks in medical equipment. In July 2015, the FDA released a warning underscoring a cyberattack on the infusion pumps. After that, in 2016, the FDA released device ‘guidance’ regarding the post-market management of cyberattacks on medical equipment. The FDA mentioned that all medical equipment possesses an undeniable risk of getting attacked, but when it is confirmed that the advantages to patients prevail over the risks, then these are permitted for marketing. Even though the rise in wireless technology and software in medical equipment has the potential for cyberattacks, these traits also offer advanced health care opportunities for patients, cutting down on waiting times [1].

Therefore, IoMT devices are significantly changing the nature of healthcare—treatment, monitoring, and, hence, offering a better lifestyle. Countries around the world are facing challenges in upgrading healthcare services. However, the latest advancements in the fields of sensors, the internet, cloud computing, big data, and others offer substantial healthcare options, but these connected IoT devices are at risk of security breaches. Statistics show that device manufacturers are not focusing much on the security protocols while developing them [1].

Moreover, unauthorised access to these IoT devices can also lead to potential harm to patients’s information, health, and safety. Hence, authentication and encryption could help solve these issues. The capability to gather and represent the data by healthcare companies in a proper way will certainly affect the future of IoMT. It should be noted that IoT will not reinstate healthcare organisations, but it will help collect all the information required for medical personnel to properly diagnose and treat patients so that it can minimise problems and incompetence in the healthcare sector [2]. Table 4 summarises the details about the IoMT devices, including their features, pros, and cons.

Even though there are lots of challenges using IoMT, different use cases provide effective solutions for patients and doctors. The quality of life is also getting upgraded with the applications of IoMT. With the availability of 5G technologies, medical applications will enhance further and will be more efficient in providing remote prescriptions or even surgeries. Moreover, all the microchips are getting smaller, and with the use of nanotechnology, these medical devices using IoT platforms are also getting miniaturised. For instance, smart watches using 5G technology will be able to provide more control for patients and doctors, such as prescription updates, taking and sending photos, etc. Precisely, atrial fibrillation (AF) is one of the most common cardiac arrhythmias and the primary cause of death worldwide. Various people are living their lives with AF without knowing about the condition. Two out of three strokes can be treated when AF has been discovered and treated. Hence, there is a potential opportunity to work in this sector, where real-time tracking solutions for the AF can be further developed to avert the leading cause of deaths [1]. Furthermore, 5G technologies, with their higher data rate and lower latency, will be more effective in providing faster solutions as required. Also, the inclusion of VR with 5G will certainly help the medical teams and the patients to arrange and chat with each other virtually and even monitor and manage the entire proceedings seamlessly. Even 10 years ago, this technique was considered impossible, but with the latest advancements in technology and wireless applications, it is not farfetched anymore.

Subsequently, treating more complex diseases such as Parkinson disease, kidney failure, intestine complications, stones in the gall bladder, arms, ankles, foot, and others could be easily observed and prescribed by the doctors immediately so that patients with critical concerns and urgent needs would be apt to evaluate medical personnel without any further delay. Hence, the fields of telehealth and telemedicine will also grow to fulfil the demands of patients and health-care service providers.

Moreover, artificial intelligence could play an imperative role in the applications of IoMT. For example, continuous tracking and management of the drugs; regular AI could handle updates on the patients’ conditions; smart hospital beds; and even basic queries could be implemented by IoMT devices. Deeper analysis can also be made with the help of AI, as it can help make the decision based on the previous records and past histories of the patients and suggest alternatives to the medical personnel. This will save more time and generate smart solutions during times of need.

### 4.1. Views and the Significance of This Research

Based on the literature surveys and findings so far, as explained above, most of the reviews discussed and investigated the IoMT functionalities in detail, such as connectivity, how doctors and patients can interact constantly using IoT platforms, how the data is being handled in clouds, and different IoMT devices that are interconnected to extract and analyse the information of the patients. In contrast, the inclusion of IoMT sensors for fabrication purposes, the usage of IoMT devices for different applications, the suitability of various IoT platforms to formulate and analyse disease conditions, and the data storage process have been some of the highlights of this research. Moreover, since the pandemic situation such as COVID-19 is responsible for millions of deaths worldwide, a case study has also been considered, and the primary devices based on an ICU environment that can be connected in dire times have also been discussed. Hence, the paper serves the message that with the recent improvements, IoT technology has demonstrated that it is an integral component of day-to-day operations, and IoT will keep its users on the proper and quick processing track. IoT, in particular, in the medical industry, may make everyone’s life a lot simpler by processing and controlling the complete system in which medical equipment is connected to each other as well as accessing an online database. Doctors may verify the changes on a frequent basis and save the data, and patients can also update their ailments on a regular basis.

Subsequently, any IoMT project must include R&D, and expanding it might be the first step towards project success. The medical industry can reduce risks, optimise costs, and treat patients more efficiently by utilising a range of IoMT applications. Teams in R&D oversee both the technical and medical components of an IoMT solution. They are responsible for determining the application’s usefulness and its potential for use in the medical field. Additionally, R&D teams might look at potential opportunities in the future. The study’s findings, prototypes, and more precise guidance regarding the IoMT product should be the final output. Before it becomes a product, the R&D team can study the concept and provide assistance in moulding it into a useful tool that addresses medical problems. The target audience of this paper is the medical industry research and development department. IoMT becomes a wonderful complement to current R&D procedures as the medical industry integrates it into their operations. R&D and IoMT can work together to increase value in the following ways:

Improved ROI for R&D: In comparison to traditional methodologies, many IoT-using industries have claimed faster return on investment (ROI) on projects. Hence, medical and healthcare industry R&D experts may now concentrate on real-time process data at accuracy levels previously unattainable because of the ability to incorporate data that covers everything. Additionally, it removes most of the uncertainty from projects by allowing them to concentrate development on items that have been demanded in recent times.

Innovative Products: IoMT has the potential to offer fresh tools for R&D in the medical sector. The employment of digital twins is one such. Advanced data analytics utilise real variables to simulate a new or enhanced product in 3D and forecast wear, output, quality issues, and duration. This lowers the number of prototype iterations, thereby lowering the cost, speeding up the time to market, and enabling engineers and technicians to refine the product before prototyping. The basic capabilities of IoMT technology serve to inform and drive the R&D process for the medical industry by enabling the analysis of more precise data and the incorporation of new process enhancements into existing product lines.

New Services: IoMT applications can provide different and innovative services that are time-adjacent. And the medical industry’s R&D can incorporate IoMT and develop new medical services, providing ideas and applications for the medical industry.

Sustainability: Many industries are working to make their production methods more sustainable. While this holds true for the majority of industries, it is particularly important for sectors like the food and medical industries, where waste, spoilage, and shelf life are still big concerns. In order to identify and address the core causes of medical industry service provision and operations, it is suitable for them to utilise IoMT to establish R&D efforts that will increase sustainability in the medical industry.

Cost Reductions: Cost is, of course, a factor that every industry addresses. R&D teams have the possibility to concentrate on what generates profits and helps in greater service provision in the medical industry thanks to the ability to use real-time production data, customer feedback, and lifecycle tracking. And these automated digital technologies help to produce a more agile R&D effort when combined with the strength of sophisticated analytics, machine learning, and AI in an IoMT platform. And it can be proven that R&D, which includes IoMT in their toolkit, is responsible for the enhancement of current processes as well as the introduction of new ones optimised using IoMT data.

Therefore, IoMT devices in the clinical area have limitless possibilities and a vast gap that can be shrunk by making them a game changer in the coming years. And by incorporating IoMT, medical sector R&D can improve the functionality and adequacy of service provision in the healthcare industry. Thus, the target audience of this paper is primarily related to the R&D of the medical industry.

### 4.2. Future Aspects

The tech companies in the healthcare space are trying to focus on reducing the cost of treatment, providing much more accessible healthcare, and making data available and protected. The following digital ingenuities have reformed healthcare and will change even more in the near future [88].

Telemedicine: This process has been considered by many as a better way of handling acute diseases than regular office visits. It offers patients convenience and freedom, which means healthcare services are not limited to a certain location or office. Also, patients residing in rural areas can access the data using electronic devices. Moreover, it saves time and money and also enables quick catch-up with the doctors without physically travelling to the hospitals.

Wearables and Digital Sensors Biotelemetry: Digitalised healthcare has brought diagnostic procedures to smart phones and other wearables using advanced sensors where the user can monitor their heart rate, blood glucose levels, blood pressure, pulse, and oxygenated blood saturation levels. The most prevalent types of wearable technology are fitness-tracking bands and smart watches. Fitness bands, such as Fitbit, monitor exercise levels by measuring the number of steps taken by the user, the number of stairs climbed, and how many calories have been burned as well. Smart watches, for instance, the Apple watch, Samsung watch, etc., also now have similar capabilities and can offer near-accurate measurements.

Moreover, Biotelemetry captures patient information using digital sensors to observe vital signs, for instance, changes in the cardiac rate during a specified time frame in a day. These data can be assessed by the health professional to make decisions and to take any pre-emptive steps as required. The findings can also produce a pool of big data, which could be very useful for scientific and research purposes.

Virtual Rehabilitation: In the year 2016, the National University of Singapore initiated an IoT-oriented reformation for the patients who’ve suffered from cardiac arrest. This rehabilitation encompassed the utilisation of wearable sensors that observed the daily actions of the patients and documented the results, where the physicians directed them using a smart phone or tablet. By providing regular therapy without the need for the patients to commute to the hospital or the rehabilitation centre, it would save time and money. It also minimises the cost to the hospital of sending these healthcare professionals to the patient’s home.

Intelligent Fabric: This technology reforms workplace design by utilising artificial intelligence (AI) and virtual reality (VR). Installations are now completed much quicker using intelligent fabric, which enables self-attaching, and AI automates the auto-configuration of virtual structures and, therefore, the design of technology amenities.

Subsequently, the COVID-19 pandemic has sparked discussions about the future of IoT in healthcare and how it can safely connect healthcare professionals and patients outside of the expanding market for healthcare IoT. In order to continue treating these patients without raising their risk of infection by admitting them into care facilities, hospitals and clinics were compelled to consider telehealth right away. Additionally, hospitals are under constant pressure to find ways to save money. The quantity of resources required at the healthcare facility may be decreased by wearable technology that allows some patients to receive treatment and monitoring at home.

The advent of 5G networks, which offer 100 times faster connectivity than conventional 4G networks, is another technology influencing the future of IoT in healthcare. IoT devices depend on connectivity for data transfer and communication between the patient and the healthcare professional. IoT devices can exchange significantly larger volumes of data at a much faster rate thanks to faster cellular data transfer. With these advancements, new healthcare IoT applications include tools to help patients adhere to their medication at home; sleep monitoring tools that can track heart rate, oxygen levels, and movements for high-risk patients; tools for remote temperature monitoring; and continuous glucose monitoring sensors that connect to mobile devices and warn patients and clinicians of changing blood sugar levels.

IoT adoption will expand as a result of the recent progress and current pandemic experience, which will also persuade people to adopt the technology who would have previously shied away from it.

IoT devices are becoming smarter and doing more than just relaying data from patients to healthcare professionals, thanks to the growing use of cloud services and AI. Smart glucose monitoring systems and smart insulin pens are two examples of IoT devices that leverage cloud services for data analysis. These two technologies not only record glucose levels over time, but they also upload the information to a cloud service or mobile app for analysis. The insulin pump can then administer the correct amount of insulin to the patient in accordance with the findings of the analysis. Another illustration is the employment of sophisticated nanny cameras to keep an eye on geriatric patients. These intelligent cameras can detect routine deviations, such as when an older person uses the restroom but does not return after a brief interval. The camera can also be used to detect falls, which would notify caretakers or emergency personnel.

The use of bots or virtual agents to communicate with patients is another IoT use that will start to gain popularity in the near future. Seniors can access a personal virtual assistant to remind them to take their medications, ask them questions about their health or pain levels, and respond to any data collected from their devices, such as glucose levels, fall detection, or oxygen levels, by combining sensor data collected by various IoT devices and sensors and using voice-enabled speakers.

Healthcare firms will implement IoT in facilities for inventory management and equipment tracking, in addition to wearables and patient-specific interactions. The scale of the sensors and developments in wireless technology have led to continued advancements in this technology, also known as real-time location systems. Hospitals will have a better understanding of potential equipment shortages and who may have come into contact with the equipment by tracking the movement of equipment and its general use. This is crucial for preventing the spread of infections, as demonstrated by the COVID-19 pandemic, which made hospitals keep track of staff members and equipment that had contact with patients who were afflicted.

## 5. Conclusions

IoMT sensors and devices, with their recent advancements, have proven that they are part and parcel of day-to-day activities, and IoT will also keep its users on the right and fast processing track. Amongst different IoMT sensors and devices, a general ICU scenario has been considered, and the most common sensors and devices have been identified for this systematic review. Particularly in the medical field, IoT can make everyone’s life a lot easier by processing and managing the entire system where the medical devices will be connected to each other along with access to the online database. Moreover, doctors can check the updates regularly and store the data, and patients can update their conditions on a regular basis as well. These updates can be visualised, managed, and monitored using various IoT-based platforms suitable for medical applications, such as KHARE, Kaa, MindSphere, and so on. The doctor and checking what prescriptions have been given can bring up the past histories of any patients using such platforms. Moreover, IoT-connected hearables, contact lenses, blood labs, tracking systems, and others are changing outcomes right now. With the inclusion of 5G technology and AI, these Medical IoT devices will be even more effective and will offer significant features, notably interactive communication between patients and doctors, remote surgery, VR-assisted check-ups, and so on. Even though the devices are getting more advanced and robust these days, by employing IoT technology, especially by equipping the sensors for the medical devices, they can assess, communicate, store, and access data from any cloud-based directory. Hence, in short, IoT applications in the medical arena have substantial gaps and limitless opportunities, which could be minimised, and thereby it could be a game-changer event in the upcoming years.

## Figures and Tables

**Table 1:** Search strings used for this systematic review.

Sl No	Search ‘Strings’
1	Medical IoT
2	Internet of Things platform
3	Artificial intelligence in IoMT
4	IoMT sensors
5	COVID-19
6	IoMT devices
7	5G and cellular communication

**Table 2:** Inclusion–exclusion conditions for this systematic review.

Inclusion Criteria	Exclusion Criteria
Studies related to IoMT devices, sensors, circuit diagrams, and IoT platforms	Pilot papers and forum posts
Studies related to COVID-19, AI and cellular communication, and data management	Articles not related to cellular and AI-based IoMT devices and management

**Table 3:** COVID-19 assistance using IoMT devices, software, services, models, and networks, including features, pros, and cons.

COVID-19 IoMT Assistance	Device or Software and Services	Features	Technology	Rate of Accuracy (%)	References
Wearable strain sensor	Device	Detects the volume and rate of breathing of the user’s respiratory system.	Track and observe patients’ breathing conditions via IoMT		[94]
Wearable IoT-based stress detection device	Device	Suitable option for persons experiencing worry, anxiety, and isolation as a result of the epidemic.			[96]
Wearable light IoT devices	Device	Employ contact tracing to alleviate social distance by alerting users when they are too near to each other.	Tags are transmitted via Bluetooth technology		[97]
COVID-19 contact tracing application	Software and services	Verifies positive instances and alerts Canadians who have been affected.			[98]
Ambulance IoT-assisted technology	Software and services	Timesaving solution	Allows specialists to advise employees on important procedures to cope with patients in emergency situations		[99]
Emergency Medical Services (EMS)	Software and services	Delivers real-time information on the number of accessible beds, all forms of blood levels, blood type availability, and availability of doctors.			[100]
Faster Region CNN with ResNet101 (FRCR)	Software and services	The FRCR has a 98% accuracy rate.		98% on chest X-ray	[101,120]
Attention-based deep 3D multiple instance learning (AD3D-MIL)	Software and services	Automated screening	Bernoulli distribution of labels was used by AD3D-MIL for efficient learning	97.9%	[102,120]
COVID GAN	Software and services	i. Auxiliary classifier model. ii. Generates synthetic chest X-ray pictures. iii. Cross-validation was not present.		95%	[104,105,120]
VGG19	Software and services	i. Automated categorization method. ii. Utilised to reduce sample bias and improve image quality. iii. Data fusion strategies improve classification accuracy.	CNN-based transfer learning architecture	93%	[106,120]
3DCNN	Software and services	i. Isolates the infection locations. ii. Removes the uneven distribution of pneumonia-infected regions. iii. Precision of the affected area is still lacking.			[106,120]

**Table 4:** IoMT devices: features, pros, and cons.

IoMT Devices	Features	Technology Used	Examples	References
Hearables	i. Changed the way people suffered hearing loss. ii. Permits filtering, equalising, and inserting layered features.	Compatible with Bluetooth.	Doppler labs	[121]
Ingestible sensors	i. Pill-sized sensors. ii. Assist in curbing symptoms and offer early notification for diabetic patients.		Proteus Digital Health	[121]
Moodables	i. Mood-enhancing devices. ii. Head-mounted wearables.	Transmits a low-intensity current to the brain, which raises our mood.	Thync and Halo Neurosciences are currently working on it.	[121]
Computer vision technology	i. Drone technology with the assistance of AI. ii. Visually affected people to direct effectively.	To imitate visual insight.	Skydio uses computer vision technology.	[121]
Healthcare recording	Lessen manual work to keep a record of patients’s data.	Driven by voice instructions and obtains the patient’s information.	IoT devices, for example, Audemix.	[121]
Blood labs	Contain five sensors to trace substances in the body, e.g., glucose and lactate.	Substance tracing is analysed and can be sent via Bluetooth or cellular network.	Small implantable devices created by The Swiss Federal Institute of Technology.	[122]
Connected inhalers	Providing asthma control for the patient’s symptoms and treatment.	i. Sensor connected to an inhaler or Bluetooth spirometer. ii. Sensor connects to an app to provide details.	Smart asthma technology by Propeller Health.	[122]
Connected cancer treatment	Patients getting treatment for head and neck cancer using smart IoT technology.	i. Treatment consists of using a Bluetooth-supported weight scale and a blood pressure cuff. ii. A symptom-tracking app to send daily updates.		[122]
IoT-connected contact lenses	Non-invasive and to cure long-sightedness (presbyopia) and cataract surgery recovery.	Smart contact lenses being created.	Contact lens known as Triggerfish by Swiss company, Sensimed.	[122]
Depression monitoring	To monitor the patient’s moods and thoughts.	A smartwatch app could be utilised to evaluate the effects of depression.	Major depressive disorder (MDD) could be traced using this app.	[122]
Blood clotting	Permits patients to examine how fast the blood coagulates.	i. Bluetooth-enabled testing system. ii. Able to transmit results wirelessly.	Roche’s blood-clotting device	[122]
Connected radiology	Radiology data can be shared over the cloud-based network system so that doctors can monitor the reports remotely.	Features of the emergency room will also be connected and accessible to the doctors for testing.	Nokia’s radiology monitoring system	[123]
Closed-loop insulin distribution	glucose checking and on-demand insulin distribution for diabetic patients.	Is a sensor-based wearable with data and glucose management capability and an insulin dosage counter and delivery	Bigfoot	[123]
Tracking system	Indoor positioning and monitoring, asset tracking, and even infection monitoring.	Uses ultrasound technology.	Sonitor	[123]

## Data Availability

Not applicable.

## References

[1] Venkatesh A. IoT Connected Medical Devices Promise a Better Tomorrow. 8 February 2018. https://www.biospectrumasia.com/analysis/27/10292/iot-connected-medical-devices-promise-a-better-tomorrow.html.

[2] Patientmd (2018). IoT in Healthcare: Scope, Future and Challenges. https://steemit.com/health/@patientmd/iot-in-healthcare-scope-future-and-challenges.

[3] Park H., Kim H., Joo H., Song J. (2016). Recent advancements in the Internet-of-Things related standards: A oneM2M perspective. ICT Express.

[4] Miorandi D., Sicari S., De Pellegrini F., Chlamtac I. (2012). Internet of things: Vision, applications and research challenges. Ad Hoc Netw..

[5] Azzawi M.A., Hassan R., Bakar K.A. (2016). A Review on Internet of Things (IoT) in Healthcare. https://www.semanticscholar.org/paper/A-Review-on-Internet-of-Things-(-IoT-)-in-Azzawi-Hassan/da6188fb4af608fc8d7961eb0634b35ad81ba53d.

[6] Marketresearch.com IoT in Healthcare Market by Component (Medical Device, Systems & Software, Services, and Connectivity Technology), Application (Telemedicine, Connected Imaging, and Inpatient Monitoring), End User and Region-Global Forecast to 2028. https://www.marketresearch.com/MarketsandMarkets-v3719/IoT-Healthcare-Component-Medical-Device-33871518/.

[7] Markets & Markets IoT Healthcare Market by Component (Medical Device, Systems & Software, Services, and Connectivity Technology), Application (Telemedicine, Connected Imaging, and Inpatient Monitoring), End User and Region-Global Forecast to 2028. IoT in Healthcare Market. https://www.marketsandmarkets.com/Market-Reports/iot-healthcare-market-160082804.html.

[8] Healthcare G. Bringing the Internet of Things to Healthcare. Medical Device Network. https://www.medicaldevice-network.com/comment/bringing-internet-things-healthcare/.

[9] Yan Y., Li Q., Li H., Zhang X., Wang L. (2013). A home-based health information acquisition system. Health Inf. Sci. Syst..

[10] Darwish S., Nouretdinov I., Wolthusen S.D. (2017). Towards Composable Threat Assessment for Medical IoT (MIoT). Procedia Comput. Sci..

[11] Kitchenham B., Charters S. (2007). Guidelines for performing Systematic Literature Reviews in Software Engineering. Keele University and Durham University Joint Report, Technical Report EBSE 2007-001. https://www.elsevier.com/__data/promis_misc/525444systematicreviewsguide.pdf.

[12] Page M.J., McKenzie J.E., Bossuyt P.M., Boutron I., Hoffmann T.C., Mulrow C.D., Shamseer L., Tetzlaff J.M., Akl E.A., Brennan S.E. (2021). The PRISMA 2020 statement: An updated guideline for reporting systematic reviews. BMJ.

[13] Vimbi V., Shaffi N., Mahmud M., Subramanian K., Hajamohideen F. (2023). Application of Explainable Artificial Intelligence in Alzheimer’s Disease Classification: A Systematic Revie. https://www.researchsquare.com/article/rs-2734771/v1.

[14] Haddaway N.R., Page M.J., Pritchard C.C., McGuinness L.A. (2022). PRISMA2020: An R package and Shiny app for producing PRISMA 2020-compliant flow diagrams, with interactivity for optimised digital transparency and Open Synthesis. Campbell Syst. Rev..

[15] Cooking Hacks MySignals SW Complete Kit (eHealth Medical Development Platform) at MG Super Labs India. https://www.mgsuperlabs.co.in/estore/MySignals-SW-Complete-Kit-eHealth-Medical-Development-Platform?search=MySignals%20SW%20eHealth.

[16] Talati D. Understanding the Working of Embedded IoT Medical Devices-DZone. https://dzone.com/articles/understanding-the-working-of-embedded-iot-medical.

[17] Buda R.A., Addi M.M. A portable non-invasive blood glucose monitoring device. Proceedings of the 2014 IEEE Conference on Biomedical Engineering and Sciences (IECBES).

[18] Nguyen Gia T., Dhaou I.B., Ali M., Rahmani A.M., Westerlund T., Liljeberg P., Tenhunen H. (2019). Energy efficient fog-assisted IoT system for monitoring diabetic patients with cardiovascular disease. Future Gener. Comput. Syst..

[19] Bhat G.M., Bhat N.G. A novel IoT based framework for blood glucose examination. Proceedings of the 2017 International Conference on Electrical, Electronics, Communication, Computer, and Optimization Techniques (ICEECCOT).

[20] Gia T.N., Ali M., Dhaou I.B., Rahmani A.M., Westerlund T., Liljeberg P., Tenhunen H. (2017). IoT-based continuous glucose monitoring system: A feasibility study. Procedia Comput. Sci..

[21] Sargunam B., Anusha S. IoT Based Mobile Medical Application for Smart Insulin Regulation. Proceedings of the 2019 IEEE International Conference on Electrical, Computer and Communication Technologies (ICECCT).

[22] Istepanian R.S.H., Hu S., Philip N.Y., Sungoor A. The potential of Internet of m-health Things “m-IoT” for non-invasive glucose level sensing. Proceedings of the 2011 Annual International Conference of the IEEE Engineering in Medicine and Biology Society.

[23] Walia M.S. Temperature Detector System|Detailed Circuit Diagram Available. https://www.electronicsforu.com/electronics-projects/0-100c-temperature-detector.

[24] Ota H., Chao M., Gao Y., Wu E., Tai L.-C., Chen K., Matsuoka Y., Iwai K., Fahad H.M., Gao W. (2017). 3D Printed “Earable” Smart Devices for Real-Time Detection of Core Body Temperature. ACS Sens..

[25] Pradhan B., Bhattacharyya S., Pal K. (2021). IoT-Based Applications in Healthcare Devices. J. Healthc. Eng..

[26] Robert Blood Pressure Sensor Circuit. http://www.seekic.com/circuit_diagram/sensor_circuit/pressure_sensor/blood_pressure_sensor_circuit.html.

[27] Fu Y., Liu J. (2015). System Design for Wearable Blood Oxygen Saturation and Pulse Measurement Device. Procedia Manuf..

[28] Phan D.T., Phan T.T.V., Huynh T.C., Park S., Choi J., Oh J. (2022). Noninvasive, Wearable Multi Biosensors for Continuous, Long-term Monitoring of Blood Pressure via Internet of Things Applications. Comput. Electr. Eng..

[29] Lingaiah T.B., Kumar D.H., Nagaraja C. (2013). Measurement of Pulse rate and SPo2 using Pulse Oximeter developed using LabVIEW. IOSR J. Electr. Electron. Eng..

[30] Agustine L., Muljono I., Angka P.R., Gunadhi A., Lestariningsih D., Weliamto W.A. Heart Rate Monitoring Device for Arrhythmia Using Pulse Oximeter Sensor Based on Android. Proceedings of the 2018 International Conference on Computer Engineering, Network and Intelligent Multimedia (CENIM).

[31] Phan D.T., Nguyen C.H., Nguyen T.D.P., Tran L.H., Park S., Choi J., Lee B., Oh J. (2022). A Flexible, Wearable, and Wireless Biosensor Patch with Internet of Medical Things Applications. Biosensors.

[32] Technology P. Electrocardiogram (ECG) Circuit Diagram for Use with Oscilloscopes. https://www.picotech.com/library/application-note/electrocardiogram-ecg-circuit-for-use-with-oscilloscopes.

[33] Tekeste T., Saleh H., Mohammad B., Ismail M. (2019). Ultra-Low Power QRS Detection and ECG Compression Architecture for IoT Healthcare Devices. IEEE Trans. Circuits Syst. I Regul. Pap..

[34] Bathilde J.B., Then Y.L., Chameera R., Tay F.S., Zaidel D.N.A. Continuous heart rate monitoring system as an IoT edge device. Proceedings of the 2018 IEEE Sensors Applications Symposium (SAS).

[35] Drew B.J., Califf R.M., Funk M., Kaufman E.S., Krucoff M.W., Laks M.M., Macfarlane P.W., Sommargren C., Swiryn S., Van Hare G.F. (2004). Practice standards for electrocardiographic monitoring in hospital settings: An American Heart Association scientific statement from the Councils on Cardiovascular Nursing, Clinical Cardiology, and Cardiovascular Disease in the Young: Endorsed by the International Society of Computerized Electrocardiology and the American Association of Critical-Care Nurses. Circulation.

[36] Dash P.K. (2002). Electrocardiogram Monitoring. Indian J. Anaesth..

[37] Agu E., Pedersen P., Strong D., Tulu B., He Q., Wang L., Li Y. The smartphone as a medical device: Assessing enablers, benefits and challenges. Proceedings of the 2013 IEEE International Workshop of Internet-of-Things Networking and Control (IoT-NC).

[38] Liu M.L., Tao L., Yan Z. (2012). Internet of Things-based electrocardiogram monitoring system. Chin. Pat..

[39] Wu T., Redouté J.-M., Yuce M., Fortino G., Wang Z. (2019). A Wearable, Low-Power, Real-Time ECG Monitor for Smart T-shirt and IoT Healthcare Applications. Advances in Body Area Networks I.

[40] Cotter N.E., Christensen D., Furse K. Electromyogram Circuit. https://www.coursehero.com/file/20565460/ECE1270-Lab1b/.

[41] Güler İ., Baştürk N., Samutoğlu N., Küçük K. Real-Time Abnormal Detection for Asthma Patients with Internet of Things Technology. Proceedings of the 2018 3rd International Conference on Computer Science and Engineering (UBMK).

[42] Li B., Dong Q., Downen R.S., Tran N., Jackson J.H., Pillai D., Zaghloul M., Li Z. (2019). A wearable IoT aldehyde sensor for pediatric asthma research and management. Sens. Actuators B Chem..

[43] Gurbeta L., Badnjevic A., Maksimovic M., Omanovic-Miklicanin E., Sejdic E. (2018). A telehealth system for automated diagnosis of asthma and chronical obstructive pulmonary disease. J. Am. Med. Inform. Assoc..

[44] Shah S.T.U., Badshah F., Dad F., Amin N., Ahmad Jan M. (2019). Cloud-Assisted IoT-Based Smart Respiratory Monitoring System for Asthma Patients. Applications of Intelligent Technologies in Healthcare.

[45] Raji A., Kanchana Devi P., Golda Jeyaseeli P., Balaganesh N. Respiratory monitoring system for asthma patients based on IoT. Proceedings of the 2016 Online International Conference on Green Engineering and Technologies (IC-GET).

[46] Khaliq A., Awan M., UmairSaleh M., Abbas M., Khan S. (2014). Real Time Wireless Health Monitoring System. IOSR J. Electr. Electron. Eng..

[47] Alam M.G.R., Abedin S.F., Moon S.I., Hong C.S. CNN based Mood Mining through IoT-based Physiological Sensors Observation. Proceedings of the 2017 Korean Computer Science and Technology Conference, Korean Society for Information Science.

[48] Ahmad E. (2020). Meezaj: An Interactive System for Real-Time Mood Measurement and Reflection based on Internet of Things. Int. J. Adv. Comput. Sci. Appl..

[49] Pandey P.S. Machine Learning and IoT for prediction and detection of stress. Proceedings of the 2017 17th International Conference on Computational Science and Its Applications (ICCSA).

[50] Dhipa M., Priyadharshini M.K.S., Vinovarthini S., Swetha N. (2023). Depression Mood Detector. Int. Res. J. Mod. Eng. Technol. Sci..

[51] MySignals MySignals SW Technical Guide. https://development.libelium.com/mysignals/documentation/mysignals-sw-technical-guide.

[52] ScreenCloud 15 IoT Applications in the Connected Healthcare Space-ScreenCloud. https://screencloud.com/blog/healthcare/iot-applications-healthcare-space.

[53] QardioCore Smart Wearable ECG EKG Monitor-QardioCore. Qardio. https://www.qardio.com/qardiocore-wearable-ecg-ekg-monitor-iphone/.

[54] Dsouza A. Fewer Doctors, More Freedom, Greater Health. Zanthion: Vanguards of Technology-driven Senior Care System. Healthcare Tech Outlook. https://www.healthcaretechoutlook.com/zanthion.

[55] Hou B., Yi L., Hu D., Luo Z., Gao D., Li C., Xing B., Wang J.-W., Lee C.N., Zhang R. (2023). A swallowable X-ray dosimeter for the real-time monitoring of radiotherapy. Nat. Biomed. Eng..

[56] He R., Liu H., Niu Y., Zhang H., Genin G.M., Xu F. (2022). Flexible Miniaturized Sensor Technologies for Long-Term Physiological Monitoring. NPJ Flex. Electron..

[57] Aldeer M., Javanmard M., Martin R. (2018). A Review of Medication Adherence Monitoring Technologies. Appl. Syst. Innov..

[58] Latif G., Shankar A., Alghazo J.M., Kalyanasundaram V., Boopathi C.S., Arfan Jaffar M. (2020). I-CARES: Advancing health diagnosis and medication through IoT. Wirel. Netw..

[59] Sahlab N., Jazdi N., Weyrich M., Schmid P., Reichelt F., Maier T., Meyer-Philippi G., Matschke M., Kalka G., Ahram T., Karwowski W., Pickl S., Taiar R. (2020). Development of an Intelligent Pill Dispenser Based on an IoT-Approach. Human Systems Engineering and Design II.

[60] Shreyas A.R., Sharma S., Shivani H., Sowmyarani C.N., Shetty N.R., Patnaik L.M., Nagaraj H.C., Hamsavath P.N., Nalini N. (2019). IoT-Enabled Medicine Bottle. Emerging Research in Computing, Information, Communication and Applications.

[61] Bharadwaj S.A., Yarravarapu D., Reddy S.C.K., Prudhvi T., Sandeep K.S.P., Reddy O.S.D. Enhancing healthcare using m-care box (monitoring non-compliance of medication). Proceedings of the 2017 International Conference on Innovative Mechanisms for Industry Applications (ICIMIA).

[62] Medina J., Espinilla M., García-Fernández Á.L., Martínez L. (2018). Intelligent multi-dose medication controller for fever: From wearable devices to remote dispensers. Comput. Electr. Eng..

[63] Ghorbel A., Amor N.B., Jallouli M. (2019). A survey on different human-machine interactions used for controlling an electric wheelchair. Procedia Comput. Sci..

[64] Carrasquilla-Batista A., Quiros-Espinoza K., Gomez-Carrasquilla C. An Internet of Things (IoT) application to control a wheelchair through EEG signal processing. Proceedings of the 2017 International Symposium on Wearable Robotics and Rehabilitation (WeRob).

[65] DSouza D.J., Srivastava S., Prithika R. (2019). IoT based Smart Sensing Wheelchair to Assist in Healthcare. Int. Res. J. Eng. Technol..

[66] Ramya K.M., Nargees S., Tabasuum S.A., Khan S. (2020). A survey on Smart automated WheelChair system with voice controller using IOT along with health monitoring for physically challenged persons. Int. Sci. J. Contemp. Res. Eng. Sci. Manag..

[67] Lee Y.K., Lim J.M., Eu K.S., Goh Y.H., Tew Y. Real time image processing based obstacle avoidance and navigation system for autonomous wheelchair application. Proceedings of the 2017 Asia-Pacific Signal and Information Processing Association Annual Summit and Conference (APSIPA ASC).

[68] Ghorbel A., Bouguerra S., Amor N.B., Jallouli M. Cloud based mobile application for remote control of intelligent wheelchair. Proceedings of the 2018 14th International Wireless Communications & Mobile Computing Conference (IWCMC).

[69] Onasanya A., Elshakankiri M. (2021). Smart integrated IoT healthcare system for cancer care. Wirel. Netw..

[70] Lapresa M., Tamantini C., Scotto Di Luzio F., Cordella F., Bravi M., Miccinilli S., Zollo L. A Smart Solution for Proprioceptive Rehabilitation through M-IMU Sensors. Proceedings of the 2020 IEEE International Workshop on Metrology for Industry 4.0 & IoT.

[71] Adamovich S.V., Merians A.S., Boian R., Lewis J.A., Tremaine M., Burdea G.S., Recce M., Poizner H. (2005). A Virtual Reality—Based Exercise System for Hand Rehabilitation Post-Stroke. Presence Teleoperators Virtual Environ..

[72] Qi J., Yang P., Waraich A., Deng Z., Zhao Y., Yang Y. (2018). Examining sensor-based physical activity recognition and monitoring for healthcare using Internet of Things: A systematic review. J. Biomed. Inform..

[73] Nave C., Postolache O. Smart Walker based IoT Physical Rehabilitation System. Proceedings of the 2018 International Symposium in Sensing and Instrumentation in IoT Era (ISSI).

[74] Yang G., Deng J., Pang G., Zhang H., Li J., Deng B., Pang Z., Xu J., Jiang M., Liljeberg P. (2018). An IoT-Enabled Stroke Rehabilitation System Based on Smart Wearable Armband and Machine Learning. IEEE J. Transl. Eng. Health Med..

[75] Heshmat M., Shehata A.-R.S. A Framework about Using Internet of Things for Smart Cancer Treatment Process. Proceedings of the International Conference on Industrial Engineering and Operations Management.

[76] Rajan J.P., Rajan S.E., Martis R.J., Panigrahi B.K. (2020). Fog Computing Employed Computer Aided Cancer Classification System Using Deep Neural Network in Internet of Things Based Healthcare System. J. Med. Syst..

[77] Pradhan K., Chawla P. (2020). Medical Internet of things using machine learning algorithms for lung cancer detection. J. Manag. Anal..

[78] Liu Z., Yao C., Yu H., Wu T. (2019). Deep reinforcement learning with its application for lung cancer detection in medical Internet of Things. Future Gener. Comput. Syst..

[79] Rodrigues D.D.A., Ivo R.F., Satapathy S.C., Wang S., Hemanth J., Filho P.P.R. (2020). A new approach for classification skin lesion based on transfer learning, deep learning, and IoT system. Pattern Recognit. Lett..

[80] Phan D.T., Ta Q.B., Ly C.D., Nguyen C.H., Park S., Choi J., Se H.O., Oh J. (2023). Smart Low Level Laser Therapy System for Automatic Facial Dermatological Disorder Diagnosis. IEEE J. Biomed. Health Inform..

[81] Phan D.T., Mondal S., Tran L.H., Thien V.T.M., Nguyen H.V., Nguyen C.H., Park S., Choi J., Oh J. (2021). A flexible, and wireless LED therapy patch for skin wound photomedicine with IoT-connected healthcare application. Flex. Print. Electron..

[82] Phan D.T., Ta Q.B., Huynh T.C., Vo T.H., Nguyen C.H., Park S., Choi J., Oh J. (2021). A smart LED therapy device with an automatic facial acne vulgaris diagnosis based on deep learning and internet of things application. Comput. Biol. Med..

[83] Cecil J., Gupta A., Pirela-Cruz M., Ramanathan P. (2018). An IoMT based cyber training framework for orthopedic surgery using Next Generation Internet technologies. Inform. Med. Unlocked.

[84] Su H., Ovur S.E., Li Z., Hu Y., Li J., Knoll A., Ferrigno G., De Momi E. Internet of Things (IoT)-based Collaborative Control of a Redundant Manipulator for Teleoperated Minimally Invasive Surgeries. Proceedings of the 2020 IEEE International Conference on Robotics and Automation (ICRA).

[85] Bhatia K., Singh M. (2019). Towards development of portable instantaneous smart optical device for hemoglobin detection non invasively. Health Technol..

[86] Carroll N. (2016). Key Success Factors for Smart and Connected Health Software Solutions. Computer.

[87] Howard S. Integrating Smart Technology into Medical Devices. https://rainrfid.org/integrating-smart-technology-into-medical-devices/.

[88] Medical Tourism Magazine Trends in Healthcare Digital Revolution. https://www.magazine.medicaltourism.com/article/trends-healthcare-digital-revolution.

[89] Kaa Start Building Your Solution with Kaa Enterprise IoT Platform. https://www.kaaiot.com.

[90] Kaa What Is an IoT Platform? Kaa IoT platform. https://www.kaaiot.com/blog/what-is-iot-platform.

[91] Singh R.P., Javaid M., Haleem A., Suman R. (2020). Internet of things (IoT) applications to fight against COVID-19 pandemic. Diabetes Metab. Syndr. Clin. Res. Rev..

[92] Zhou W., Jia Y., Peng A., Zhang Y., Liu P. (2019). The Effect of IoT New Features on Security and Privacy: New Threats, Existing Solutions, and Challenges Yet to Be Solved. IEEE Internet Things J..

[93] Western Shelter COVID-19 Screening, Monitoring, and Isolation Part 1—Western Shelter. https://westernshelter.com/blog/2020/8/3/covid-19-screening-monitoring-and-isolation.

[94] Uday S., Jyotsna C., Amudha J. Detection of Stress using Wearable Sensors in IoT Platform. Proceedings of the 2018 Second International Conference on Inventive Communication and Computational Technologies (ICICCT).

[95] Singh S., Hamidon M.N., Zuber M., Ahmad K.A. (2021). Wireless Sensing Technology with IoMT approach for Continuous Monitoring of Breathing Rate and Volume during COVID-19. Front. Sustain. Cities.

[96] Technologies T. “Proximity Trace”, Triax Technologies. https://www.triaxtec.com/resource/fact-sheet/proximity-trace/.

[97] Alberta COVID-19 info for Albertans. https://www.alberta.ca/coronavirus-info-for-albertans.aspx.

[98] Kamal M., Aljohani A., Alanazi E. (2020). IoT meets COVID-19: Status, Challenges, and Opportunities. arXiv.

[99] Isabella A., Lekshmi K.S., Thamizhvaani E.P., Vishali S. (2018). IOT Based Emergency Medical Services. Int. J. Eng. Tech..

[100] Ahmed I., Ahmad A., Jeon G. (2021). An IoT-Based Deep Learning Framework for Early Assessment of Covid-19. IEEE Internet Things J..

[101] Lutkevich B., DelVecchio A. Internet of Medical Things (IoMT) or Healthcare IoT. https://www.techtarget.com/iotagenda/definition/IoMT-Internet-of-Medical-Things.

[102] Waheed A., Goyal M., Gupta D., Khanna A., Al-Turjman F., Pinheiro P.R. (2020). CovidGAN: Data Augmentation Using Auxiliary Classifier GAN for Improved Covid-19 Detection. IEEE Access.

[103] Ouyang X., Huo J., Xia L., Shan F., Liu J., Mo Z., Yan F., Ding Z., Yang Q., Song B. (2020). Dual-Sampling Attention Network for Diagnosis of COVID-19 from Community Acquired Pneumonia. arXiv.

[104] Horry M.J., Chakraborty S., Paul M., Ulhaq A., Pradhan B., Saha M., Shukla N. (2020). COVID-19 Detection Through Transfer Learning Using Multimodal Imaging Data. IEEE Access.

[105] Chowdhury M.E.H., Rahman T., Khandakar A., Mazhar R., Kadir M.A., Mahbub Z.B., Islam K.R., Khan M.S., Iqbal A., Emadi N.A. (2020). Can AI Help in Screening Viral and COVID-19 Pneumonia?. IEEE Access.

[106] Zheng S., Yang L., Zhou P., Li H., Liu F., Zhao R. (2021). Recommendations and guidance for providing pharmaceutical care services during COVID-19 pandemic: A China perspective. Res. Soc. Adm. Pharm..

[107] Yang T., Gentile M., Shen C.-F., Cheng C.-M. (2020). Combining Point-of-Care Diagnostics and Internet of Medical Things (IoMT) to Combat the COVID-19 Pandemic. Diagnostics.

[108] Gupta R., Misra A. (2020). Contentious issues and evolving concepts in the clinical presentation and management of patients with COVID-19 infectionwith reference to use of therapeutic and other drugs used in Co-morbid diseases (Hypertension, diabetes etc). Diabetes Metab. Syndr. Clin. Res. Rev..

[109] Ghosh A., Gupta R., Misra A. (2020). Telemedicine for diabetes care in India during COVID19 pandemic and national lockdown period: Guidelines for physicians. Diabetes Metab. Syndr. Clin. Res. Rev..

[110] Du Q., Song H., Zhu X. (2019). Social-Feature Enabled Communications Among Devices Toward the Smart IoT Community. IEEE Commun. Mag..

[111] Partila P., Tovarek J., Ilk G.H., Rozhon J., Voznak M. (2020). Deep Learning Serves Voice Cloning: How Vulnerable Are Automatic Speaker Verification Systems to Spoofing Trials?. IEEE Commun. Mag..

[112] Han Z., Wei B., Hong Y., Li T., Cong J., Zhu X., Wei H., Zhang W. (2020). Accurate Screening of COVID-19 Using Attention-Based Deep 3D Multiple Instance Learning. IEEE Trans. Med. Imaging.

[113] Qian X., Fu H., Shi W., Chen T., Fu Y., Shan F., Xue X. (2020). M^3Lung-Sys: A Deep Learning System for Multi-Class Lung Pneumonia Screening From CT Imaging. IEEE J. Biomed. Health Inform..

[114] Liao I. 5 Real-Time & Remote Patient Monitoring Trends. https://www.mpo-mag.com/contents/view_online-exclusives/2018-08-22/5-real-time-remote-patient-monitoring-trends/.

[115] Budko D. The Change of Healthcare Industry and Modern IT Trends: Where Do We Stand Now? HackerNoon.com. https://medium.com/hackernoon/the-change-of-healthcare-industry-and-modern-it-trends-where-do-we-stand-now-d5c834ae2a13.

[116] ReportLinker Internet of Things (IoT) in Healthcare. https://www.reportlinker.com/p05881047/Internet-of-Things-IoT-in-Healthcare.html.

[117] Noppitak S., Surinta O. (2021). Ensemble Convolutional Neural Network Architectures for Land Use Classification in Economic Crops Aerial Images. ICIC Express Lett..

[118] Sakib S., Tazrin T., Fouda M.M., Fadlullah Z.M., Guizani M. (2020). DL-CRC: Deep Learning-Based Chest Radiograph Classification for COVID-19 Detection: A Novel Approach. IEEE Access.

[119] Rahman A., Hossain M.S., Alrajeh N.A., Alsolami F. (2021). Adversarial Examples—Security Threats to COVID-19 Deep Learning Systems in Medical IoT Devices. IEEE Internet Things J..

[120] Iskanderani A.I., Mehedi I.M., Aljohani A.J., Shorfuzzaman M., Akther F., Palaniswamy T., Latif S.A., Latif A., Alam A. (2021). Artificial Intelligence and Medical Internet of Things Framework for Diagnosis of Coronavirus Suspected Cases. J. Healthc. Eng..

[121] Pisuwala U. Internet of Things in Healthcare: Apps, Benefits, & Challenges. https://www.peerbits.com/blog/internet-of-things-healthcare-applications-benefits-and-challenges.html.

[122] MasterAdminStrate Iot applications in Healthcare. https://www.strate.education/gallery/news/healthcare-iot.

[123] Digiteum Team Internet of Medical Things (IomT) Solutions for Medical Device Development. Digiteum. https://www.digiteum.com/internet-medical-things-medical-software-development/.

